# Neuroprotective Potential of Major Alkaloids from *Nelumbo nucifera* (Lotus): Mechanisms and Therapeutic Implications

**DOI:** 10.3390/ijms26178280

**Published:** 2025-08-26

**Authors:** Douyang Zhao, Linlin Ma, Jeremy Brownlie, Kathryn Tonissen, Yang Pan, Yunjiang Feng

**Affiliations:** 1 Institute for Biomedicine and Glycomics, Griffith University, Brisbane 4111, Australia; douyang.zhao@griffithuni.edu.au (D.Z.); linlin.ma@griffith.edu.au (L.M.); k.tonissen@griffith.edu.au (K.T.); 2 School of Environment and Science, Griffith University, Brisbane 4111, Australia; j.brownlie@griffith.edu.au; 3 School of Pharmacy, Nanjing University of Chinese Medicine, Nanjing 210023, China; ypan@njucm.edu.cn

**Keywords:** *Nelumbo nucifera*, lotus alkaloids, neuroprotection, calcium signaling, neurogenesis, neurotransmitter modulation, oxidative stress, Alzheimer’s disease, Parkinson’s disease

## Abstract

*Nelumbo nucifera* (lotus) has long been used in traditional medicine across Asia, and its bioactive alkaloids have recently garnered attention for their neuroprotective properties. This review summarizes the current research on the mechanisms by which lotus-derived alkaloids, particularly neferine, nuciferine, liensinine, and isoliensinine, protect neural tissues. These compounds exhibit a wide range of pharmacological activities, including antioxidant and anti-inflammatory effects, regulation of calcium signaling and ion channels, promotion of neurogenesis, and modulation of key neurotransmitter systems, such as dopaminergic, cholinergic, and GABAergic pathways. Notably, they attenuate tau hyperphosphorylation, reduce oxidative stress-induced neuronal apoptosis, and enhance neurotrophic signaling via BDNF-related pathways. While antioxidant and anti-inflammatory actions are the most extensively studied, emerging evidence also highlights their roles in autophagy modulation and mitochondrial protection. Together, these findings suggest that lotus alkaloids are promising candidates for the prevention and treatment of neurodegenerative diseases, such as Alzheimer’s and Parkinson’s diseases. Further investigation is warranted to explore the synergistic mechanisms and potential clinical applications of these compounds.

## 1. Introduction

**Figure 1:**
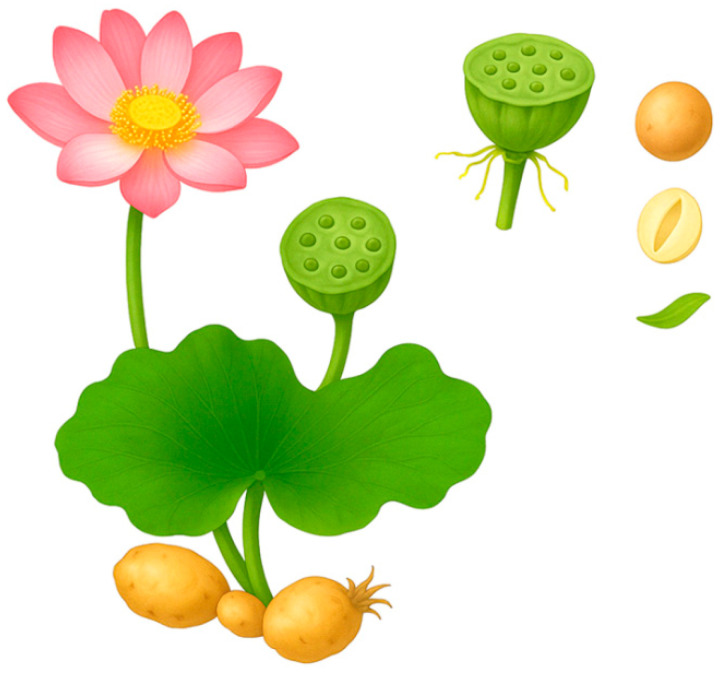
Major morphological features of lotus (*Nelumbo nucifera*) used in traditional medicine. This anatomical illustration shows the key structural components of the lotus plant used for medicinal purposes, specifically the flower, broad leaves, rhizome structures that form root nodules, and circular seedpod. To the right are enlarged images of the key reproductive structures: (top) the mature seed with the epicarp (external layer) intact; (middle) a horizontal cross-section of the seed showing the large starch-rich cotyledon surrounding a cavity that encases the plumule; and (bottom) the plumule, which is the developmental core from which the embryo develops.

Traditional Chinese Medicine (TCM) has utilized botanical remedies for thousands of years, with plant-derived compounds playing a central role in the treatment of various ailments [1]. *Nelumbo nucifera*, commonly known as the Indian lotus, padma, Chinese water lily, and sacred lotus, is a perennial aquatic plant of the family Nymphaeaceae [2,3]. Although the family contains only one genus (Nelumbo), it comprises two species: *N. nucifera Gaertn.* (Asian lotus) and *N. lutea Pear.* (American lotus). *N. nucifera* is widely distributed across China, Thailand, India and Japan [4]. The majority of *N. nucifera* parts are edible, but its rhizomes and seeds are the most commonly used in traditional diets [5,6]. Lotus rhizomes, which are morphologically modified underground stems, are typically yellow and have a stout and creeping appearance [7,8]. Lotus seeds typically have an oval or round shape, measuring approximately 1.2 to 1.8 cm in length and 0.8 to 1.4 cm in diameter, with an average weight ranging from 1.1 to 1.4 g (Figure 1) [5]. The plant also features large, floating, orbicular leaves that can reach diameters of up to 60 cm [2,9]. While the rhizome and seeds are valued for their nutritional content, the lotus flower is often used for decorative purposes due to its low nutritional value [7,10].

Historically, lotus seeds have been used in TCM to treat a range of conditions, including hypertension, nausea, vomiting of blood, red eyes, and swelling [11,12]. Their medicinal use was first documented in the Shi Xing Ben Cao (Edible Materia Medica) during the Tang dynasty (circa 1400 AD), and they have been used continuously for over 400 years in China [4]. The Ben Cao Gang Mu General Outline (Compendium of Materia Medica, 1596) noted that the lotus embryo helps clear internal heat, calm the mind, and promote homeostasis [13]. In Yi Lin Zuan Yao (1647), a soup prepared from lotus embryos and Ba-Ceng-Sha was described as a remedy for purifying the heart and kidneys [4]. Modern applications continue to emphasize their calming effects, including the treatment of insomnia, anxiety, and restlessness [14,15]. Lotus seed tea, especially when combined with goji berries, is also promoted for enhancing cognitive function, calming the nervous system, and improving sleep quality [16,17].

Beyond the embryo and seeds, other parts of *N. nucifera*, notably the leaves and rhizomes, are widely used in traditional medicine [18,19]. Lotus leaves have been employed to regulate cholesterol levels, alleviate summer heat-induced dehydration, and treat diarrhea caused by damp-heat, spleen deficiency, and hematochezia [20]. They are also believed to enhance cerebral blood circulation and support cognitive functions [21,22]. Lotus rhizomes are traditionally used for chronic diseases, such as cancer and diabetes, and have been shown to exhibit hepatoprotective, cardioprotective, and hypoglycemic effects [23,24,25]. When consumed as lotus root soup, they are thought to boost energy, promote circulation, and support overall brain health [14,15,26].

The primary pharmacological activity of *N. nucifera* is largely attributed to its alkaloid content. Preliminary studies have identified several bioactive alkaloids, including nuciferine, neferine, and liensinine, which modulate key molecular pathways implicated in neurodegeneration, thereby demonstrating their potential as neuroprotective agents. Recent research has shown that neferine can attenuate oxidative stress and preserve neuronal integrity [27,28,29,30,31]. Additionally, emerging evidence suggests that these alkaloids may influence dopaminergic and serotonergic signaling pathways, offering therapeutic potential for alleviating anxiety, insomnia, and other neuropsychiatric symptoms [32,33].

While both traditional use and modern studies underscore the broad pharmacological potential of Nelumbo nucifera, most contemporary research has concentrated on its anti-cancer and cardioprotective effects. In this review, we have placed greater emphasis on a comprehensive perspective while acknowledging the more specific focus of previous reviews, including that of Ren et al. [34]. Furthermore, despite accumulating evidence supporting the neuroprotective potential of lotus alkaloids, a comprehensive synthesis of their pharmacological properties, molecular mechanisms, and therapeutic applications in neurodegenerative disorders remains lacking [35]. This review aims to address this gap by systematically evaluating the current body of research on alkaloids derived from *N. nucifera*, emphasizing their neuroprotective effects. Through this analysis, we aim to provide a more coherent understanding of their potential in preventing or ameliorating neurological damage and identify key directions for future investigations.

## 2. Method and Materials

An extensive literature search was conducted using Google Scholar with the keyword “lotus alkaloid neuroprotection,” which initially yielded 1810 results. To refine the scope, the search was restricted to publications from 2014 to 2024, narrowing the dataset to 1570 papers. Only peer-reviewed journal articles published in English were included, further narrowing the pool of publications to around 1000 papers. Abstracts were screened for relevance, resulting in approximately 500 papers selected for full-text assessment. Ultimately, only manuscripts aligned with the review’s objectives and providing substantive data were retained for analysis. To further refine the literature pool, additional keyword combinations were employed, including “lotus alkaloid oxidation neuroprotection,” “lotus alkaloid neuroinflammation,” “lotus alkaloid autophagy neuroprotection,” “lotus alkaloid neurogenesis,” and “lotus alkaloid mitochondria neuroprotection”. These searches helped identify studies that specifically addressed the mechanistic underpinnings of neuroprotection by lotus-derived alkaloids. Based on these selection criteria, 173 peer-reviewed articles were ultimately included and cited in this review.

## 3. Chemical Constituents of Lotus Alkaloids

**Figure 2:**
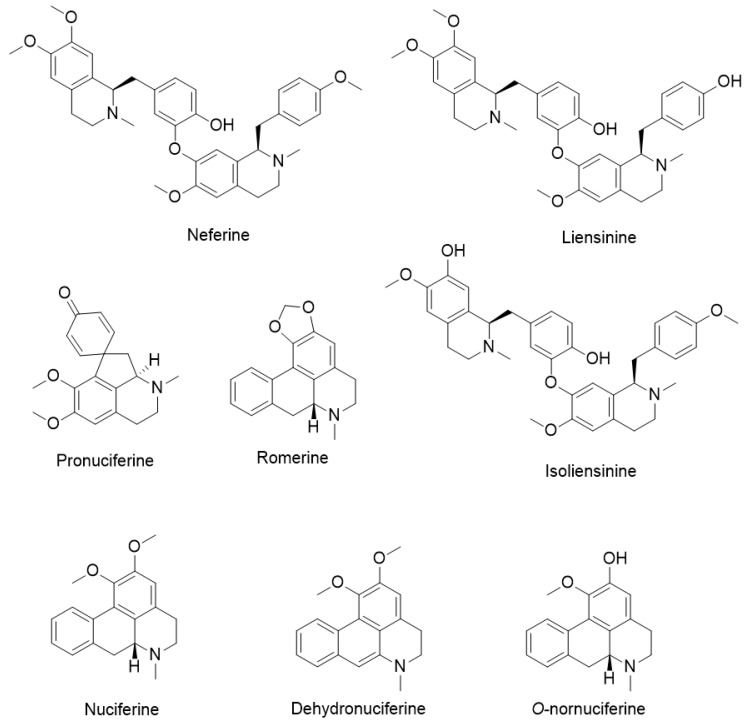
Key bioactive alkaloids present in *Nelumbo nucifera* with neuroprotective effects.

Alkaloids are the major secondary metabolites in Lotus, accounting for approximately 2.43% of its dry weight [27,28,29]. To date, 51 alkaloids, divided into four classes, have been identified, including 1-benzylisoquinoline (Figure S1), aporphines (Figure S2), bisbenzylisoquinolines (Figure S3), and tribenzylisoquinolines (Figure S4), with more than half belonging to the isoquinoline class [4,36]. Neferine, liensinine, isoliensinine, nuciferine, *O*-nornuciferine, dehydronuciferine, pronuciferine, and roemerine are recognized as key bioactive alkaloids present in *Nelumbo nucifera*, which have been demonstrated in the present study to exert neuroprotective effects (Figure 2). Neferine, liensinine, and isoliensinine are classified as bisbenzylisoquinoline alkaloids, whereas nuciferine, *O*-nornuciferine, dehydronuciferine, pronuciferine, and roemerine belong to the aporphine alkaloid class. In the present review, their neuroprotective effects will be discussed in detail within the mechanism section, with an emphasis on their roles in modulating neurodegenerative diseases. For clarity, these eight alkaloids are highlighted as the primary focus of discussion, while the chemical structures of the other alkaloids are provided in the Supplementary Materials to improve readability.

## 4. Mechanism of Neuroprotection

Several mechanisms have been demonstrated to be involved in neuroprotection, including anti-inflammation, autophagy regulation, antioxidation, mitochondrial regulation, ion channel regulation, and neurogenesis (Table 1, Table 2, Table 3, Table 4 and Table 5). These mechanisms can work synergistically to enhance neuroprotection, although their interactions are complex and remain to be fully elucidated. In this section, we discuss how lotus alkaloids protect neuronal cells through these mechanisms and explore the interplay between them.

### 4.1. Anti-Inflammatory Effects and Autophagy Regulation

Inflammation is a natural defense mechanism that facilitates the clearance of cellular debris after injury or necrosis [37]. However, prolonged or excessive neuroinflammation may induce cerebrum damage and cerebrovascular dysfunction, ultimately triggering neuronal apoptosis [38]. Moreover, the resulting inflammatory cascade further propagates neuroinflammation, creating a self-amplifying cycle [37,39]. Chronic neuroinflammation is a hallmark of neurodegenerative diseases, particularly Parkinson’s disease [40,41].

Microglia, the resident immune cells of the CNS, play a pivotal role in neuroinflammation. Although they act as the first line of defense, their overactivation may result in the progression of Parkinson’s disease [40]. One key player in this process is inducible nitric oxide synthase (iNOS), which catalyzes the production of nitric oxide (NO) and is a major mediator of inflammation [42]. Overexpression of iNOS leads to excessive NO production, resulting in tissue damage and chronic inflammation [42]. In vivo studies have demonstrated that lotus alkaloids suppress iNOS activity. Liensinine (20 mg/kg and 40 mg/kg) significantly reduced iNOS levels and cerebral inflammation in mice [43]. Similarly, neferine (10 μM) suppressed iNOS expression and the release of pro-inflammatory cytokines, such as interleukin-6 (IL-6) and tumor necrosis factor-alpha (TNF-α), in LPS-stimulated BV-2 microglial cells [40], supporting its potent anti-inflammatory effects (Table 1).

**Table 1:** Neuroprotective effects of lotus alkaloids via anti-inflammatory mechanisms. ↓ indicates decrease.

Compound	Dose	Model	Mechanism	Reference(s)
Neferine	10 µM	LPS-treated BV-2 cells	↓ INOS ↓ interleukin-6 ↓ TNFα	[40,44]
Nuciferine	5–20 μM	LPS-stimulated BV2 microglia cells	↓ IL-1β ↓ PGE2 ↓ TNFα ↓ NO secretion ↓ NF-κB	[45]
Liensinine	20 and 40 mg/kg	Sepsis-associated encephalopathy mice cerebrum	↓ iNOS activities ↓ inflammatory responses	[43,46]
Liensinine, neferine, and isoliensinine	5.02 μM, 4.13 μM and 4.36 µM (*IC*_50_ values)	Mouse macrophage cell line	↓ NO production ↓ NF-κB ↓ IL-1β ↓ PGE2 ↓ TNFα	[47]

The anti-inflammatory effects of lotus alkaloids, including those described above, could involve NF-κB signaling, an important transcription factor that upregulates the expression of genes involved in stimulating inflammation, such as TNF-α, IL-1β, PGE2 (Prostaglandin E2), and iNOS expression (Table 1) [48]. NF-κB can be activated by various mechanisms, but one common mechanism is via phosphorylation and subsequent degradation of IκBα (inhibitor of kappa B alpha), an inhibitor protein that binds to the NF-κB transcription factor and prevents its translocation to the nucleus [49].

**Figure 3:**
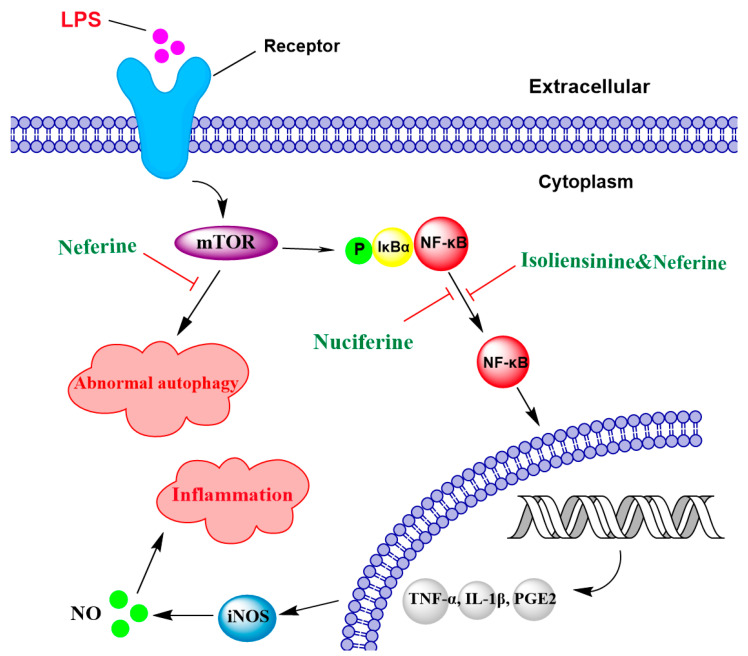
Lotus alkaloids exert neuroprotection by regulating inflammation triggered by LPS. Nuciferine, neferine, and isoliensinine attenuate inflammatory responses by inhibiting the phosphorylation and subsequent proteasomal degradation of inhibitor of kappa B alpha (IκBα), thereby suppressing the activation of the NF-κB signaling pathway. This results in the downregulation of inducible nitric oxide synthase (iNOS) expression and inflammatory mediators, including TNF-α, IL-1β, PGE2, and NO production, thereby decreasing inflammation. Furthermore, neferine inhibits mechanistic Target of Rapamycin (mTOR) activation, thereby reducing autophagy dysfunction.

NF-κB pathway activation can be simulated by many factors, including LPS, which results in the rapid secretion of cytokines and other pro-inflammatory mediators [48]. Treatment of BV2 microglial cells with 5–20 μM nuciferine significantly decreased the secretion of LPS-induced inflammatory mediators, including TNF-α, IL-1β, and PGE2, by inhibiting IκBα phosphorylation, thus preventing the activation of NF-κB (Figure 3) [45,46]. Treatment of BV2 cells with 10 μM neferine also inhibited the expression of LPS-induced inflammatory cytokines, including TNF-α and IL-6 [40]. Liensinine, neferine, and isoliensinine at 5.02 µM, 4.13 µM, and 4.36 µM (IC50 values) were also shown to inhibit LPS-induced NO production in the RAW 264.7 mouse macrophage cell line, and to suppress the release of inflammatory markers such as TNF-α, IL-1β, and IL-6 by suppressing the NF-κB pathway [47]. Therefore, the main lotus alkaloids may act by inhibiting the NF-κB signaling pathway to downregulate the expression of inflammatory mediators.

Autophagy is a fundamental catabolic process essential for neuronal homeostasis, mediating the clearance of dysfunctional organelles and protein aggregates. Its regulatory role in neuroimmune signaling, particularly in suppressing excessive neuroinflammation through modulation of inflammasome activity and cytokine release, is critical for CNS integrity. Impairment of autophagy is increasingly implicated in the pathogenesis of neurodegenerative diseases, underscoring its potential as a therapeutic target for conditions such as Alzheimer’s and Parkinson’s diseases [50,51]. Additionally, autophagy modulates cytokine production and facilitates the resolution of inflammation. The formation of autophagosomes contributes to the clearance of pro-inflammatory stimuli, thereby mitigating sustained neuroinflammation [51,52,53,54].

Dysregulation of autophagy plays a central role in the development of neurological disorders and is associated with depression [55]. *LC3B-II* and *Beclin-1* are critical proteins involved in autophagy [56,57]. Previous studies have demonstrated that total alkaloids from lotus embryos increased autophagy by significantly increasing the expression of *LC3B-II* and *Beclin-1* in the hippocampus of LPS-treated mice (200 mg kg^−1^) and BV2 microglial cells (20 μM) (Table 2) [58]. Previous studies have suggested that endoplasmic reticulum (ER) stress is closely associated with the development of depression-like behaviors induced by various physiological and environmental stressors [59]. According to the same study, total alkaloid extracts reduced LPS-induced depressive behavior by decreasing ER stress and increasing autophagy. Based on these findings, the authors proposed that consuming lotus embryo tea may be a promising strategy for preventing depression [58,59].

**Table 2:** Neuroprotective effects of lotus alkaloids via autophagy regulation. ↓ indicates decrease while ↑ suggests increase.

Compound	Dose	Model	Mechanism	Reference
Neferine	12.8 µM	PC-12 cells	↓ 50% level of Huntingtin Protein	[60]
Liensinine	100 µM	Aβ transgenic GMC101 nematodes	↑ autophagy-related genes ↑ autophagosome formation	[61]
Total alkaloids of lotus embryo	200 mg kg^−1^ 20 μM	LPS-treated mice BV2 microglial cell	↑ autophagy ↑ LC3B-II and Beclin-1 ↓ depression	[58]

Defects in autophagy are commonly observed in various chronic inflammatory diseases [62,63]. Impaired autophagic mechanisms can lead to the accumulation of damaged cellular components, thereby exacerbating chronic inflammation [63,64,65]. Therefore, autophagy regulation may offer a novel approach for modulating neurodegenerative disorders by clearing aggregate-prone proteins [66]. For example, neferine (12.8 µM) can clear 50% of Huntingtin protein level through an autophagy-related gene 7 (ATG7)-dependent mechanism [60]. This effect was mediated through the mTOR-AMPK-dependent pathway (Figure 3), a key signaling pathway that upregulates autophagy-related genes [67,68,69]. Similarly, in a transgenic nematode model of Alzheimer’s disease that overexpressed Amyloid-beta (Aβ) proteins, liensinine (100 µM) significantly decreased the accumulation of Amyloid-beta and tau proteins, thereby inhibiting abnormal autophagosome formation and avoiding subsequent apoptosis [61]. This effect was linked to liensinine’s ability to upregulate autophagy genes, including *lgg-1*, *unc-51*, *pha-4*, *atg-9*, and *ced-9* [61,70,71].

Inflammation and autophagy are linked cellular responses to stress and are required for maintaining cellular homeostasis. Inflammation can stimulate autophagy processes [72], which, in turn, can reduce inflammation and protect the cells. Conversely, during periods of chronic inflammation, autophagy is impaired, leading to increased inflammation and cellular damage [65,73]. Maintaining a balance between autophagy and inflammation is essential for overall health and recovery from infection or injury. Given the promising effects of lotus alkaloid extracts on both autophagy and inflammation, these compounds are increasingly being considered as effective therapeutics for treating a range of neurological and neurodegenerative conditions.

### 4.2. Oxidative Stress Protection and Mitochondrial Function Regulation

Oxidative stress can be harmful to sugars, lipids, proteins, and DNA, triggering neuroinflammation and leading to significant neuronal cell injury [74,75]. Excessive accumulation of reactive oxygen species (ROS) may result in hyperoxia—a state of elevated oxygen levels—which can be even more harmful, promoting neurotoxicity and ultimately inducing neuronal apoptosis [76,77]. The brain is especially vulnerable to ROS due to its high demand for oxygen [78]. Therefore, overproduction of ROS has been increasingly associated with aging and age-related diseases [79,80]. Oxidative stress is closely associated with mitochondrial dysfunction. Accumulated oxidative stress can lead to mitochondrial dysfunction, which in turn triggers irreversible mutations and dysfunction [81,82]. Mitochondria, which utilize nearly 85% of the oxygen in cells, are the primary sites of ROS production [83]. Most ROS are generated by “leakage” of electrons from the mitochondrial respiratory chain [83,84], leading to the formation of incompletely reduced oxygen species such as hydrogen peroxide (H_2_O_2_), the radical anion O_2_^−^ radical, and the nitric oxide radical (NO•) [78,83]. They are highly reactive radicals that can impose severe oxidative stress on cells [78,85].

**Figure 4:**
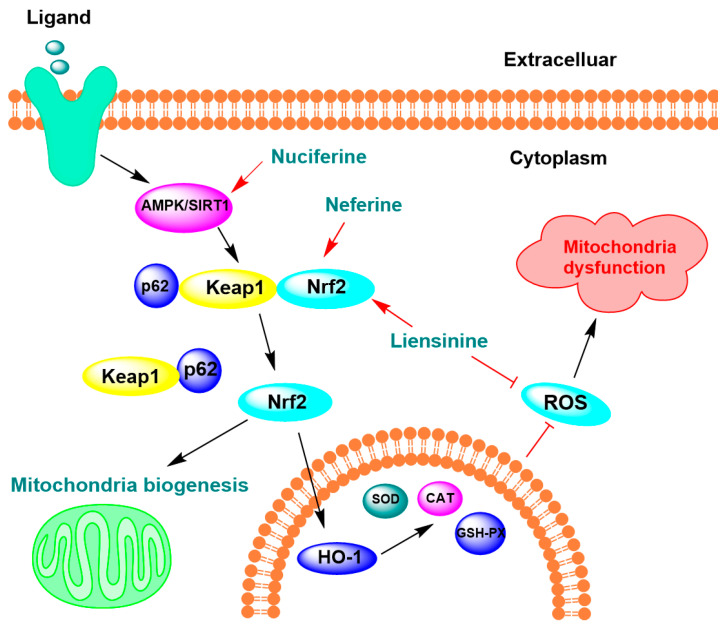
Lotus alkaloids exert neuroprotection by regulating oxidation and mitochondrial function. Nuciferine, neferine, and liensinine exert antioxidant effects by activating the AMP-activated protein kinase/Sirtuin 1 (AMPK/SIRT1) signaling pathway, which promotes the removal of Keap1/p62 and further nuclear translocation of nuclear factor erythroid-related factor 2 (Nrf2), thereby activating mitochondrial biogenesis. Simultaneously, this activation enhances the transcriptional upregulation of antioxidant enzymes, including heme oxygenase-1 (HO-1), catalase (CAT), superoxide dismutase (SOD), and glutathione peroxidase (GSH-Px), ultimately leading to a reduction in intracellular reactive oxygen species (ROS) levels.

Lotus alkaloids have demonstrated antioxidant defense against ROS in neuronal cells (Table 3). In an LPS-activated microglial cell model, liensinine, neferine, and isoliensinine scavenged 50% ·OH at concentrations of 5.4, 6.9, and 6.6 μM, and 50% ONOO− radicals at 10.5, 7.8, and 10.3 μM [44]. In SH-SY5Y neuroblastoma cells, 10 µM liensinine and neferine decreased ROS levels by 15.31% and 20.37%, respectively [61]. Another study on SH-SY5Y cells showed that pronuciferine at 5 and 10 µM significantly suppressed neuronal death caused by H_2_O_2_ and increased SH-SY5Y cell proliferation by 45% [86]. CAT (catalase), superoxide dismutase (SOD), and glutathione peroxidase (GSH-Px) are key antioxidant enzymes that protect cells from oxidative stress by neutralizing reactive oxygen species (ROS) [87,88]. In a high-fat diet-induced obese mouse model, nuciferine at 40 mg/kg ameliorated high-fat diet-induced insulin resistance and increased the levels of antioxidant enzymes SOD, CAT, and GSH-Px (Figure 4) [89]. This effect was attributed to the activation of the AMPK/SIRT1 pathway. According to two studies, activation of the AMPK/SIRT1 pathway reduces Aβ1-42 accumulation and phosphorylated tau (p-tau) expression, which in turn increases the levels of antioxidant enzymes [87,90]. To summarize, several lotus alkaloids exhibit potent antioxidant properties that protect neurons from oxidative stress by scavenging ROS and modulating key antioxidant enzymes.

**Table 3:** Neuroprotective effects of lotus alkaloids via antioxidation. ↑ indicates increase while ↓ suggests decrease.

Compound	Dose	Model	Mechanism	Reference
Pronuciferine	5 µM	SH-SY5Y cells	↓ neuronal death caused by H_2_O_2_, ↑ cell proliferation by 45%	[86]
Liensinine, neferine, and isoliensinine	5.4, 6.9, and 6.6 μM	LPS-activated microglial cells	↓ 50% ·OH	[44]
	10.5, 7.8, and 10.3 μM		↓ 50% ONOO−	
Liensinine and neferine	10 µM	APP695swe SH-SY5Y cells	↑ 15.31% oxidative stress resistance ↓ 20.37% ROS levels	[61]
Nuciferine	40 mg/kg	High-fat diets obese mice	↓ 69.55% GSH, ↓ 60.05% SOD ↓ 3.59% CAT	[89]

Mitochondria are essential organelles that produce energy in the form of adenosine triphosphate (ATP) through oxidative phosphorylation [91]. The brain uses approximately 25% of the body’s total energy, which is primarily supplied by mitochondria [80]. The brain and neurons depend heavily on ATP production, which also results in the creation of ROS [92,93,94]. Overproduction of ROS can lead to mitochondrial dysfunction, affecting ATP production and further increasing ROS generation, creating a vicious cycle [95,96]. Mitochondrial dysfunction has been implicated in many neurodegenerative diseases, including Alzheimer’s, Parkinson’s, and Huntington’s diseases [97]. This dysfunction often includes an impaired mitochondrial membrane potential, reduced ATP production, and increased ROS generation [98,99]. Lotus alkaloids have been shown to promote mitochondrial function (Table 4). In PC12 cells, 10 µM neferine increased the mitochondrial membrane potential and ATP levels and decreased mitochondrial ROS levels [100]. In an induced cerebral ischemia mouse model, neferine (50 mg/kg) improved mitochondrial structure, respiration, and Na^+^-K^+^-ATPase activity. This protective effect was linked to the activation of the p62–Keap1–Nrf2 pathway (Figure 4) by stimulating the nuclear translocation of Nrf2 (a transcription factor), which further activates its downstream heme oxygenase-1 (HO-1) and other antioxidant genes [100]. This highlights the critical role of oxidative stress reduction in preserving mitochondrial integrity.

**Table 4:** Neuroprotective effects of lotus alkaloids via mitochondria regulation. ↑ indicates increase while ↓ suggests decrease.

Compound	Dose	Model	Mechanism	Reference
Liensinine	40 mg/kg	Sepsis-associated encephalopathy mice	↓ cerebrum mitochondria apoptosis	[43]
Neferine	50 mg/kg	Rats with induced cerebral ischaemia	↑ mitochondrial structures ↑ mitochondrial respiration	[100]
	10 µM	PC12 cells	↑ mitochondrial membrane potentials ↓ mitochondrial ROS	

Furthermore, mitochondria are highly susceptible not only to oxidative damage but also to the activation of apoptotic signaling pathways. *Bax* and *Bcl-2* are pivotal regulators of mitochondrial-mediated apoptosis, with *Bax* promoting cell death in response to stress, and Bcl-2 protecting cells by inhibiting pro-apoptotic signals [101,102]. Liensinine at 40 mg/kg attenuated mitochondrial apoptosis in the cerebrum of sepsis-associated encephalopathy (SAE) mice by upregulating *BCL-2* expression and downregulating Bax expression, thereby inhibiting mitochondrial apoptosome formation [43]. Consistent with previous studies, this study demonstrated that the reduction in oxidative stress in SAE mice was associated with liensinine-induced upregulation of Nrf2, which promoted its nuclear translocation and subsequently enhanced the transcription of downstream antioxidant enzymes. These findings emphasize the critical role of lotus alkaloids in alleviating oxidative stress and mitochondrial dysfunction through the activation of the Nrf2 pathway [43,103].

To conclude, several studies have demonstrated that lotus alkaloids exert protective effects against oxidative stress and mitochondrial dysfunction, primarily through the activation of the Nrf2 signaling pathway [43,100]. Oxidative stress and mitochondrial dysfunction are closely interconnected, with each capable of exacerbating the other. When mitochondrial function is compromised, ROS generation increases, which in turn exacerbates mitochondrial damage, creating a vicious cycle [95,104]. Therefore, mitochondrial dysfunction and abnormalities in antioxidants can increase oxidative stress and lead to neuronal death. Lotus alkaloids that target both mitochondrial regulation and enhance antioxidant defenses are promising candidates for neuroprotection.

### 4.3. Regulation of Ion Channels

Oxidative stress not only damages proteins, lipids, DNA, and cellular signaling molecules, but also impairs ion channel function [105]. Ion channels, which are transmembrane proteins that selectively conduct Na^+^, K^+^, Ca^2+^, and Cl^−^ ions, play an important role in brain homeostasis [106,107]. Dysfunction of these channels, such as altered ion permeability or selectivity, contributes to neurodegeneration by interacting with multiple pathogenic mechanisms. For example, disrupted balance of ion homeostasis can promote excitotoxicity and mitochondrial dysfunction; loss of membrane potential can affect neuronal firing and signal transmission, impairing brain network activity; ion dysregulation can alter protein folding and degradation pathways, exacerbating the accumulation of toxic aggregates like α-synuclein or amyloid-β (Aβ); and ion channel dysfunction in glial cells can trigger neuroinflammation [108,109,110].

**Figure 5:**
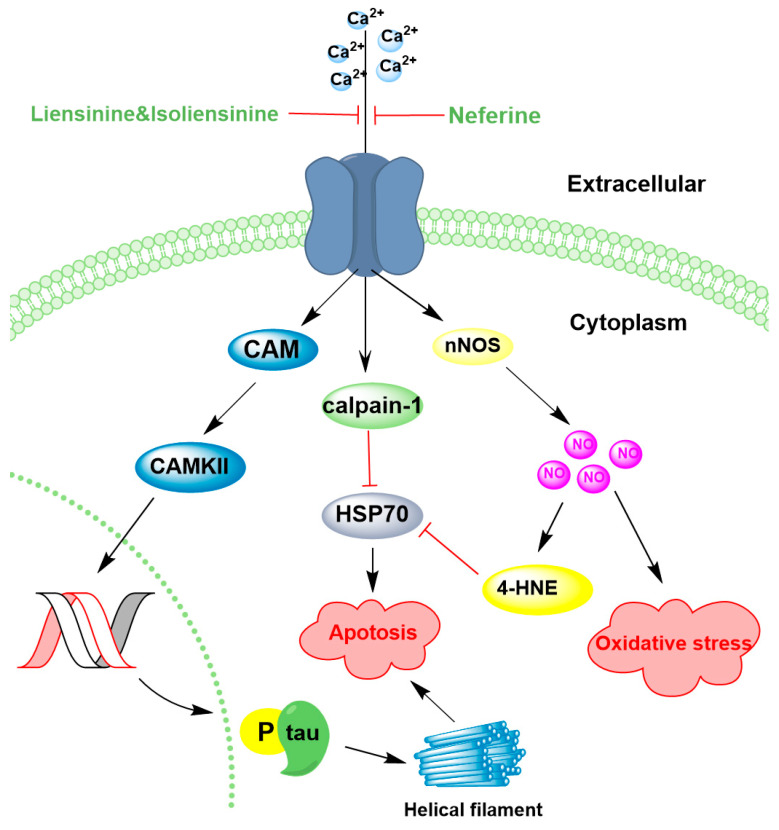
Lotus alkaloids exert neuroprotective effects by regulating calcium homeostasis. Ca^2+^ overload can be significantly reduced by liensinine and isoliensinine, which downregulate the Calcium–Calmodulin/Calcium–Calmodulin-Dependent Protein Kinase II (Ca^2+^-CaM/CaMKII) and inhibit tau protein hyperphosphorylation, thereby decreasing the formation of helical filaments. Neferine also attenuates intracellular Ca^2+^ accumulation and upregulates Hsp70 (heat shock protein 70 kDa) expression by downregulating calcium-dependent cysteine protease (calpain-1), thereby inhibiting apoptosis. Furthermore, the downregulation of neuronal nitric oxide (nNOS) simultaneously reduces 4-hydroxynonenal (4-HNE) and nitric oxide (NO) production, thereby alleviating oxidative stress.

Calcium signaling is especially vital in neurons. While it plays a crucial role in neuroprotection, dysregulated calcium influx can lead to excitotoxicity, oxidative stress, and neurodegeneration, which are implicated in conditions like Alzheimer’s and Parkinson’s diseases [111,112]. Ca^2+^ overload through over-activated calcium channels induced by Aβ can lead to a cascade of ROS-mediated oxidative stress, resulting in cerebral neuronal loss [113,114]. Notably, it was found Ca^2+^ overload in Aβ_25–35_-treated PC12 cells was significantly reduced to 72.8%, 46.9%, and 81.7% by liensinine, isoliensinine, and neferine (10 μM), respectively (Table 5) [115,116]. This protective effect was attributed to the inhibition of tau protein hyperphosphorylation by downregulating the Ca^2+^-CaM/CaMKII pathway (Figure 5) [115,117]. Hyperphosphorylation of tau protein in the brain results in the formation of neurofibrillary tangles, which are a hallmark of Alzheimer’s disease and other tauopathies, ultimately resulting in cerebral neuronal loss [116,118].

**Table 5:** Neuroprotective effects of lotus alkaloids via ion-channel modulation. ↑ indicates increase while ↓ suggests decrease.

Compound	Dose	Model	Mechanism	Reference
Neferine, Liensinine, Isoliensinine	10 μM	PC12 cells damaged by Aβ_25–35_	↓ Ca^2+^ level 72.8% and 46.9%	[115]
Neferine	50 mg/kg	Permanent middle cerebral artery occlusion (pMCAO) rats	↓ Ca^2+^ ↑ Hsp70 ↓ NO	[119]

Neuronal nitric oxide synthase (nNOS) is a key enzyme responsible for the production of nitric oxide (NO) in neurons and plays crucial roles in neurotransmission, synaptic plasticity, and vascular regulation. However, dysregulated nNOS activity, particularly in response to calcium overload, can contribute to neurodegeneration [120,121]. Intracellular Ca^2+^ accumulation is a major trigger for nNOS activation, leading to excessive NO production and oxidative stress (Figure 5). Elevated Ca^2+^ levels are associated with increased neurotoxicity and dysfunction of heat shock protein 70 kDa (Hsp70) [122]. Hsp70, which is localized in the endoplasmic reticulum and mitochondria, plays a crucial role in protein folding, cellular stress protection, and apoptosis inhibition [119,123]. A study in permanent middle cerebral artery occlusion (pMCAO) rats demonstrated that neferine (50 mg/kg) reduced intracellular Ca^2+^ levels and upregulated Hsp70 expression. This effect was mediated by the downregulation of calpain-1 (a calcium-activated protease) and 4-HNE (a lipid peroxidation product), along with a concomitant reduction in NO production (Figure 5) [119]. Both calpain-1 and 4-HNE play significant roles in neuronal injury, particularly in calcium dysregulation and oxidative stress-related neurodegeneration [124,125]. These findings suggest that neferine mitigates calcium overload and oxidative stress by upregulating Hsp70 expression, thereby protecting against organelle damage and apoptosis [119]. Collectively, these results highlight the therapeutic potential of lotus-derived alkaloids in neurodegenerative diseases characterized by calcium dysregulation.

### 4.4. Regulation of Neurogenesis

Neurogenesis, the process of generating new neurons, declines with age [126]. While many current treatments for neurodegenerative diseases aim to prevent neuronal loss, enhancing neurogenesis offers a complementary approach to support brain repair [127,128]. Neurogenesis-related genes accelerate the differentiation of neural stem cells into new neurons [129].

**Figure 6:**
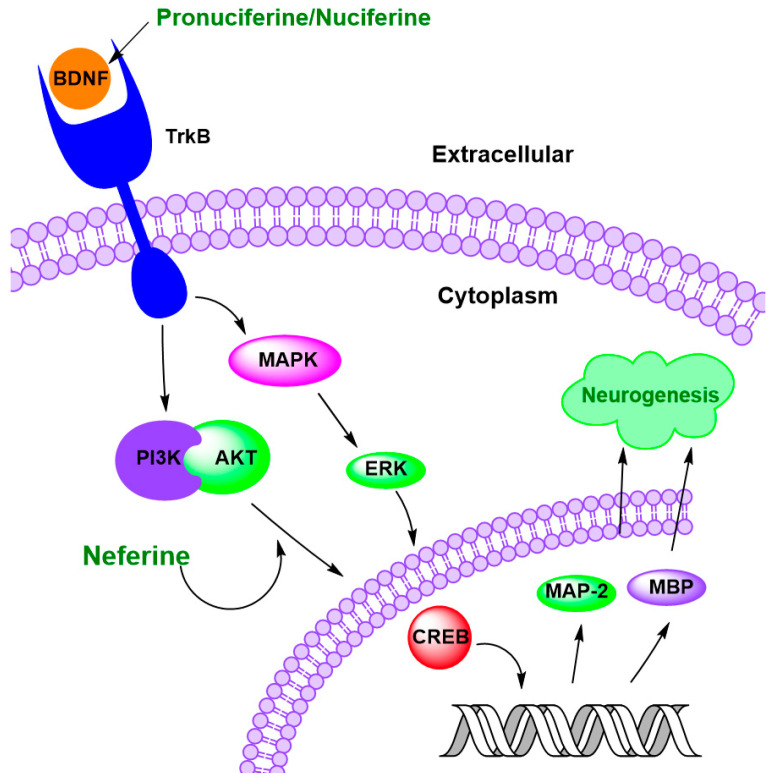
Lotus alkaloids exert neuroprotection by promoting neurogenesis. Pronuciferine and nuciferine increase brain-derived neurotrophic factor (BDNF) levels by activating the TrkB receptor, which in turn triggers key signaling pathways such as mitogen-activated protein kinase (MAPK), extracellular signal-regulated kinase (ERK), and Phosphatidylinositol 3-kinase (PI3K/Akt). This subsequently activates the transcription factor cAMP Response Element-Binding Protein (CREB), which promotes the expression of neurogenic genes, including microtubule-associated protein 2 (MAP-2) and Myelin basic protein (MBP), thereby enhancing neurogenesis.

Therefore, compounds that promote neurogenesis or enhance the expression of neurogenesis-related proteins may hold therapeutic potential for age-related neurodegenerative diseases (Table 6) [128,130]. Microtubule-associated protein 2 (MAP-2) and myelin basic protein (MBP) are essential proteins for neural development and function. MAP-2 protein stabilizes microtubules in neuronal dendrites, playing a critical role in maintaining neuronal structure and supporting dendritic growth and maturation [131,132]. In contrast, MBP protein is vital for myelin sheath formation by oligodendrocytes, ensuring proper insulation of axons and efficient nerve signal conduction in the central nervous system [133,134]. Together, they serve as functional markers of neuronal and glial cell identity and maturation. In hypoxic-ischemic-injured rats, neferine (50 mg/kg) reduced neuronal loss, promoted morphological recovery and myelination, and alleviated white matter injury in the cerebrum [135]. These effects were attributed to a significant increase in the expression of MAP-2 and MBP proteins by neferine (Figure 6) [135].

**Table 6:** Neuroprotective effects of lotus alkaloids via promotion of neurogenesis. ↑ indicates increase while ↓ suggests decrease.

Compound	Dose	Model	Mechanism	Reference
Neferine	50 mg/kg	Neonatal hypoxic-ischemic-injured rats	↓ neuronal loss, ↑ morphological recovery of the brain ↑ MAP-2 and MBP ↑ myelination	[135]
*N. nucifera* leaf water extracts	10 to 20 μg/mL	Scopolamine-treated mice	↑ hippocampus neurogenesis	[136]
Pronuciferine	0.1 to 10 μM	SH-SY5Y cells	↑ 17% to 20% BDNF level	[86]
Roemerine	20 mM and 10 mM	SH-SY5Y cells	↑ 73% and 36% BDNF expression	[137]

Brain-derived neurotrophic factor (BDNF) is a major neurotrophic factor involved in neurogenesis, which plays an important role in developing and maintaining neuronal structures, neuronal differentiation, survival, and synaptic plasticity [138,139,140,141]. Nuciferine (10 or 20 μg/mL) has been shown to promote hippocampus neurogenesis in scopolamine-treated mice by elevating BDNF level and restoring the expression of neurogenesis-associated markers, including DCX, nestin, and NeuN, markers of early, mid, and late stages of neuronal development, respectively, in the hippocampus [136]. Similarly, pronuciferine (0.1–10 μM) increased BDNF levels in SH-SY5Y cells by 17% to 20% [86]. These effects were associated with the activation of key signaling pathways, MAPK/ERK and PI3K/Akt by BDNF, ultimately leading to the activation of the transcription factor CREB (cAMP Response Element-Binding Protein) (Figure 6) [136]. CREB is a critical regulator of genes involved in neuronal survival, growth, synaptic plasticity, and neurogenesis [142,143]. Therefore, BDNF serves not only as a neurotrophic support molecule but also as a powerful modulator of gene expression that is essential for brain development, function, and adaptation.

To conclude, lotus alkaloids, particularly neferine and nuciferine, have shown promising neurogenic potential by modulating key molecular and cellular mechanisms involved in brain development and repair. These compounds upregulated the expression of MAP-2 and MBP, indicating enhanced neuronal differentiation and myelination. Mechanistically, lotus alkaloids activate neurotrophic signaling pathways, such as BDNF/TrkB, which in turn stimulates downstream activation of the PI3K/Akt and MAPK cascades. These findings suggest that lotus alkaloids may serve as valuable neuroprotective and regenerative agents in the context of neurodegenerative diseases.

### 4.5. Multimodal Actions of Lotus Alkaloids on Neurotransmitter Systems: Possible Neuroprotective Effects

Lotus alkaloids exhibit a diverse range of neurobiological effects and interact with multiple neurotransmitter systems, including dopaminergic, GABAergic, and cholinergic pathways. Their multifaceted mechanism potentially confers enhanced neuroprotective and cognitive benefits compared to single-target botanical compounds, which may explain the diverse neurological effects observed in their applications in traditional medicines.

Lotus alkaloids offer potential therapies for neurodegenerative conditions, such as Parkinson’s disease, either by modulating dopamine receptors or by elevating intracellular dopamine levels, addressing the progressive dopamine depletion that is a central pathophysiological feature of Parkinson’s disease [144,145,146]. D2 dopamine receptor agonists are widely used in the clinical management of Parkinson’s disease due to their ability to directly stimulate postsynaptic dopamine receptors, thereby bypassing the need for endogenous dopamine synthesis and release from degenerating nigrostriatal neurons. This mechanism provides more stable dopaminergic stimulation and contributes to improved symptom control, particularly in the early stages of the disease [147,148]. Among lotus-derived alkaloids, *O*-nornuciferine has been characterized as a potent agonist of both D1 and D2 dopamine receptors, exhibiting superior binding affinity (D1 *IC*_50_ = 2.09 μM and D2 *IC*_50_ = 1.14 μM) when compared to eight other lotus-derived alkaloids evaluated (ranging from 4.01 μM to 38.47 μM) [33]. A compound that targets both D1 and D2 receptors represents a pharmacologically appealing strategy for treating Parkinson’s disease, potentially offering broader symptom relief than D2-only drugs [149]. Additionally, experimental models have demonstrated that neferine administration effectively increases dopamine concentrations within the substantia nigra in 1-methyl-4-phenyl-1,2,3,6-tetrahydropyridine (MPTP)-induced Parkinson’s disease mouse models, suggesting a direct modulatory effect on dopamine metabolism or neuroprotection of dopaminergic neurons [150].

Acetylcholinesterase inhibitors (AChEIs) represent an extensively investigated therapeutic approach for Alzheimer’s disease, based on the dual role of acetylcholinesterase (AChE) in both degrading acetylcholine and accelerating the formation of amyloid fibrils, which are critical pathological features of Alzheimer’s disease [151,152]. Phytochemical studies have identified dehydronuciferine as the most potent AChE inhibitor among eight aporphine alkaloids isolated from lotus, with an *IC*_50_ of 25 μg/mL [152]. Furthermore, both neferine and liensinine inhibited AChE activity (*IC*_50_ = 14.19 and 0.34 μM, respectively) in a rat model of AD [153]. These findings suggest that lotus alkaloids may provide cognitive benefits by preserving cholinergic neurotransmission.

In several neurodegenerative disorders, disruption of GABAergic function contributes to a constellation of symptoms, including motor dysfunction, cognitive deterioration, and seizure activity [154]. Therapeutic approaches targeting GABA receptors may modulate excessive excitatory neurotransmission and potentially alleviate symptoms of Parkinson’s disease, Alzheimer’s disease, and Huntington’s disease [155]. Traditional uses of *Nelumbo nucifera*, particularly the leaves, include calming the mind and alleviating insomnia [13]. These ethnopharmacological applications have been supported by mechanistic studies demonstrating that the total alkaloids from lotus leaves can increase GABA levels in the brain, promote Cl^−^ influx via GABA_A_ receptors, and elevate serotonin and dopamine levels. Such neurotransmitter modulation is consistent with the regulation of sleep–wake cycles and reduction of anxiety. The sedative and anxiolytic effects of these alkaloids were confirmed through behavioral assays, including open-field, light/dark box, and pentobarbital-induced sleep tests, with effects significantly attenuated by GABA_A_ receptor antagonists [21,32]. These findings suggest that lotus leaf alkaloids exert their central nervous system effects primarily via GABAergic and monoaminergic pathways, providing a pharmacological basis for their traditional use in promoting relaxation and improving sleep quality.

## 5. Conclusions and Limitations

**Figure 7:**
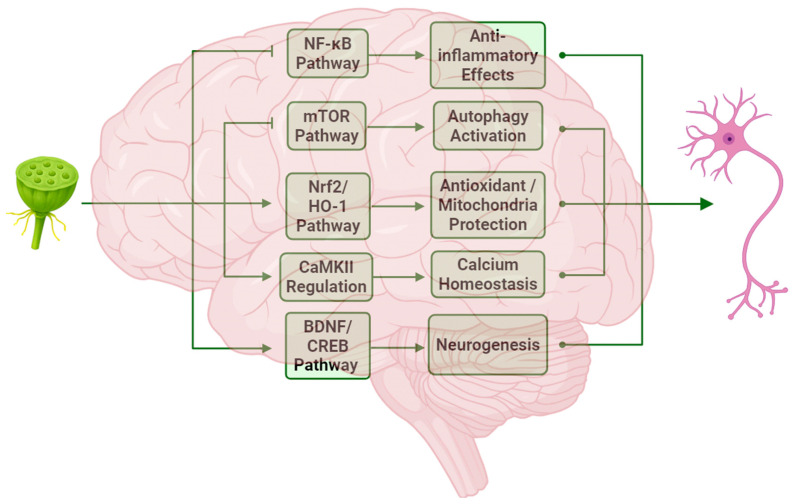
Summary of neuroprotective mechanisms of lotus alkaloids. Created in BioRender. Zhao, D. (2025) https://BioRender.com/cj686yy (accessed on 14 July 2025).

To date, 51 lotus alkaloids have been isolated; however, only a limited number have been assessed for their neuroprotective properties. Among these, neferine, liensinine, and nuciferine have demonstrated a range of neuroprotective effects through various mechanisms (Figure 7). The most common effects include anti-inflammatory and antioxidant effects, while the promotion of neurogenesis and mitochondrial regulation represents promising avenues for future research. Although the precise interactions among these mechanisms remain unclear, their interplay likely exerts a synergistic effect on neuroprotection. For instance, a strong interrelationship exists between antioxidant effects and mitochondrial regulation, while a robust association is observed between anti-inflammatory mechanisms and autophagy regulation.

Current evidence indicates that the major alkaloids of *N. nucifera* exhibit minimal cytotoxicity in a range of normal and specialized cell types. No significant toxic effects or adverse interactions have been reported at experimentally relevant concentrations. A detailed summary of the available cytotoxicity data is provided in the Supplementary Material. These findings support their favorable safety profile and justify further investigation of their pharmacological potential.

While this review highlights recent advances in understanding the neuroprotective mechanisms of lotus alkaloids, it has several limitations. The majority of evidence is derived from preclinical models, with a notable lack of human studies evaluating efficacy, safety, and pharmacokinetics. In addition, most studies investigate isolated mechanisms in controlled systems, whereas in vivo, the interplay between oxidative stress, inflammation, apoptosis, and neurogenesis is likely to be more complex. Future research should therefore prioritize translational studies, including well-designed clinical trials, structural optimization of active compounds, and integrative multi-omics approaches, to elucidate synergistic pathways and improve therapeutic potential.

## Data Availability

Not applicable.

## Supplementary Materials

The following supporting information can be downloaded at: https://www.mdpi.com/article/10.3390/ijms26178280/s1, References [156,157,158,159,160,161,162,163,164,165,166,167,168,169,170,171,172,173,174,175,176] are cited in the supplementary materials.

## Abbreviations

The following abbreviations are used in this manuscript:

Aβ	Amyloid beta
AChE	Acetylcholinesterase
AChEI	Acetylcholinesterase inhibitor
BDNF	Brain-derived neurotrophic factor
Ca^2+^	Calcium ion
CaMKII	Calcium/calmodulin-dependent protein kinase II
CAT	Catalase
CREB	cAMP response element-binding protein
D1	Dopamine freceptor D1
D2	Dopamine receptor D2
ER	Endoplasmic reticulum
GABA	Gamma-aminobutyric acid
GSH-Px	Glutathione peroxidase
Hsp70	Heat shock protein 70 kDa
HO-1	Heme oxygenase-1
IC50	Half maximal inhibitory concentration
INOS	Inducible nitric oxide synthase
IL-6	Interleukin-6
IκBα	Inhibitor of kappa B alpha
MAP-2	Microtubule-associated protein 2
MAPK	Mitogen-activated protein kinase
MBP	Myelin basic protein
MPTP	1-Methyl-4-phenyl-1,2,3,6-tetrahydropyridine
nNOS	Neuronal nitric oxide synthase
NO	Nitric oxide
Nrf2	Nuclear factor erythroid 2–related factor 2
pMCAO	Permanent middle cerebral artery occlusion
PI3K	Phosphatidylinositol 3-kinase
ROS	Reactive oxygen species
SH-SY5Y	Human neuroblastoma cell line SH-SY5Y
SOD	Superoxide dismutase
TrkB	Tropomyosin receptor kinase B
TNF-α	Tumor necrosis factor-alpha
4-HNE	4-Hydroxynonenal

## References

[1] Marshall A.C. (2020). Traditional Chinese medicine and clinical pharmacology. Drug Discovery and Evaluation: Methods in Clinical Pharmacology.

[2] Mukherjee P.K., Mukherjee D., Maji A.K., Rai S., Heinrich M. (2009). The sacred lotus (Nelumbo nucifera)—Phytochemical and therapeutic profile. J. Pharm. Pharmacol..

[3] Mukherjee P.K., Balasubramanian R., Saha K., Saha B.P., Pal M. (1996). A review on nelumbo nucifera gaertn. Anc. Sci. Life.

[4] Chen S., Li X., Wu J., Li J., Xiao M., Yang Y., Liu Z., Cheng Y. (2021). Plumula Nelumbinis: A review of traditional uses, phytochemistry, pharmacology, pharmacokinetics and safety. J. Ethnopharmacol..

[5] Zhang Y., Lu X., Zeng S., Huang X., Guo Z., Zheng Y., Tian Y., Zheng B. (2015). Nutritional composition, physiological functions and processing of lotus (Nelumbo nucifera Gaertn.) seeds: A review. Phytochem. Rev..

[6] Chen G., Zhu M., Guo M. (2019). Research advances in traditional and modern use of Nelumbo nucifera: Phytochemicals, health promoting activities and beyond. Crit. Rev. Food Sci. Nutr..

[7] Lin Z., Zhang C., Cao D., Damaris R.N., Yang P. (2019). The Latest Studies on Lotus (*Nelumbo nucifera*)—An Emerging Horticultural Model Plant. Int. J. Mol. Sci..

[8] Stubbendieck J., Milby J.L. (2021). Legumes of the Great Plains: An Illustrated Guide.

[9] Pal I., Dey P. (2015). A review on lotus (*Nelumbo nucifera*) seed. Int. J. Sci. Res..

[10] Yang H., He S., Feng Q., Liu Z., Xia S., Zhou Q., Wu Z., Zhang Y. (2024). Lotus (*Nelumbo nucifera*): A multidisciplinary review of its cultural, ecological, and nutraceutical significance. Bioresour. Bioprocess..

[11] Peng Z.Y., Zhang S.D., Liu S., He B.M. (2011). Protective effect of neferine on endothelial cell nitric oxide production induced by lysophosphatidylcholine: The role of the DDAH-ADMA pathway. Can. J. Physiol. Pharmacol..

[12] Sridhar K., Bhat R. (2007). Lotus-A potential nutraceutical source. J. Agric. Technol..

[13] Zhao X., Mu Y., Yang M. (2018). A simple multi-residue method for determination of plant growth retardants in Ophiopogon japonicus and soil using ultra-performance liquid chromatography-tandem mass spectrometry. Chemosphere.

[14] Sun W., Shahrajabian M.H., Cheng Q. (2019). Exploring Notable Functional Foods in East of Asia, Health Benefits and Ideal Nutrition. Res. Crop Ecophysiol..

[15] Shahrajabian M.H., Sun W., Cheng Q., Khoshkharam M. (2022). Exploring the quality of foods from ancient China based on traditional Chinese medicine. Res. Crop Ecophysiol..

[16] Hao W., Luo S., Hao L., Zhang F. (2023). Lotus Seedpod Oligomeric Procyanidin Nanoliposomes Targeting TLR4/NF-κB Reduce Inflammation and Oxidative Stress in Patients with Traumatic Brain Injury. J. Biomed. Nanotechnol..

[17] Chang Y., Lee J.J., Hsieh C.Y., Hsiao G., Chou D.S., Sheu J.R. (2009). Inhibitory effects of ketamine on lipopolysaccharide-induced microglial activation. Mediat. Inflamm..

[18] Bangar S.P., Dunno K., Kumar M., Mostafa H., Maqsood S. (2022). A comprehensive review on lotus seeds (Nelumbo nucifera Gaertn.): Nutritional composition, health-related bioactive properties, and industrial applications. J. Funct. Foods.

[19] Wang Y.-F., Shen Z.-C., Li J., Liang T., Lin X.-F., Li Y.-P., Zeng W., Zou Q., Shen J.-L., Wang X.-Y. (2022). Phytochemicals, biological activity, and industrial application of lotus seedpod (*Receptaculum Nelumbinis*): A review. Front. Nutr..

[20] Zheng H., Han L., Shi W., Fang X., Hong Y., Cao Y. (2022). Research advances in lotus leaf as Chinese dietary herbal medicine. Am. J. Chin. Med..

[21] Zhang H., Wu Y., Qiu M., Zheng Y., Chen L., Shi X., Yang J., Lin Q., Lin J. (2024). Lotus leaf Nuciferine improves sleep and reduces the low neuronal activity and brain tissue abnormalities associated with insomnia. Food Biosci..

[22] Temviriyanukul P., Sritalahareuthai V., Promyos N., Thangsiri S., Pruesapan K., Srinuanchai W., Nuchuchua O., Siriwan D., On-Nom N., Suttisansanee U. (2020). The effect of sacred lotus (*Nelumbo nucifera*) and its mixtures on phenolic profiles, antioxidant activities, and inhibitions of the key enzymes relevant to Alzheimer’s disease. Molecules.

[23] Zhao F., Wang F. (2024). Phytochemical Properties and Nutritional Benefits of Lotus Rhizome (*Nelumbo nucifera*): A Comprehensive Review. Int. J. Aquac..

[24] Dandin V.S., Sebastian J.K., Dalavi J.V., Nagella P., Madhav N.A., Khot V.V. (2023). Bioactive Compounds and Biological Activities of Lotus (Nelumbo nucifera Gaertn.). Bioactive Compounds in the Storage Organs of Plants.

[25] Xie J., Lin H., Jin F., Luo Y., Yang P., Song J., Yao W., Lin W., Yuan D., Zuo A. (2024). Jia Wei Qingxin Lotus Seed Drink ameliorates epithelial mesenchymal transition injury in diabetic kidney disease via inhibition of JMJD1C/SP1/ZEB1 signaling pathway. Phytomedicine.

[26] Yen C.-C., Tung C.-W., Chang C.-W., Tsai C.-C., Hsu M.-C., Wu Y.-T. (2020). Potential risk of higenamine misuse in sports: Evaluation of lotus plumule extract products and a human study. Nutrients.

[27] Arooj M., Imran S., Inam-Ur-Raheem M., Rajoka M.S.R., Sameen A., Siddique R., Sahar A., Tariq S., Riaz A., Hussain A. (2021). Lotus seeds (*Nelumbinis semen*) as an emerging therapeutic seed: A comprehensive review. Food Sci. Nutr..

[28] Wang J., Hu X., Yin W., Cai H. (1991). Alkaloids of plumula Nelumbinis. Zhongguo Zhong Yao Za Zhi.

[29] Deng X., Zhu L., Fang T., Vimolmangkang S., Yang D., Ogutu C., Liu Y., Han Y. (2016). Analysis of Isoquinoline Alkaloid Composition and Wound-Induced Variation in Nelumbo Using HPLC-MS/MS. J. Agric. Food Chem..

[30] Mitra K., Raihan M.A., Rahman M.A., Rouf R., Reza H.M., Uddin S.J., Muhammad I. (2022). Therapeutic Potential of Nelumbo nucifera (Sacred Lotus) in CNS Disorders. Dhaka Univ. J. Pharm. Sci..

[31] Manogaran P., Beeraka N.M., Padma V.V. (2019). The cytoprotective and anti-cancer potential of bisbenzylisoquinoline alkaloids from Nelumbo nucifera. Curr. Top. Med. Chem..

[32] Yan M.-Z., Chang Q., Zhong Y., Xiao B.-X., Feng L., Cao F.-R., Pan R.-L., Zhang Z.-S., Liao Y.-H., Liu X.-M. (2015). Lotus leaf alkaloid extract displays sedative–hypnotic and anxiolytic effects through GABAA receptor. J. Agric. Food Chem..

[33] Zhou H., Hou T., Gao Z., Guo X., Wang C., Wang J., Liu Y., Liang X. (2021). Discovery of eight alkaloids with D1 and D2 antagonist activity in leaves of Nelumbo nucifera Gaertn. Using FLIPR assays. J. Ethnopharmacol..

[34] Ren X., Chen H., Wang H., Wang Y., Huang C., Pan H. (2024). Advances in the pharmacological effects and mechanisms of Nelumbo nucifera gaertn. Extract nuciferine. J. Ethnopharmacol..

[35] Wang Z., Li Y., Ma D., Zeng M., Wang Z., Qin F., Chen J., Christian M., He Z. (2023). Alkaloids from lotus (Nelumbo nucifera): Recent advances in biosynthesis, pharmacokinetics, bioactivity, safety, and industrial applications. Crit. Rev. Food Sci. Nutr..

[36] Zhang H., Wang X., Guo Y., Liu X., Zhao X., Teka T., Lv C., Han L., Huang Y., Pan G. (2021). Thirteen bisbenzylisoquinoline alkaloids in five Chinese medicinal plants: Botany, traditional uses, phytochemistry, pharmacokinetic and toxicity studies. J. Ethnopharmacol..

[37] Li J., Wu Y., Dong S., Yu Y., Wu Y., Xiang B., Li Q. (2023). Research progress on neuroprotective effects of isoquinoline alkaloids. Molecules.

[38] Yang C., Hawkins K.E., Doré S., Candelario-Jalil E. (2019). Neuroinflammatory mechanisms of blood-brain barrier damage in ischemic stroke. Am. J. Physiol.-Cell Physiol..

[39] Kempuraj D., Thangavel R., Natteru P., Selvakumar G., Saeed D., Zahoor H., Zaheer S., Iyer S., Zaheer A. (2016). Neuroinflammation induces neurodegeneration. J. Neurol. Neurosurg. Spine.

[40] Li T., Zhai Y.X., Zheng T., Xu B. (2023). Neferine exerts anti-inflammatory activity in BV-2 microglial cells and protects mice with MPTP-induced Parkinson’s disease by inhibiting NF-kappaB activation. Mol. Med. Rep..

[41] Glass C.K., Saijo K., Winner B., Marchetto M.C., Gage F.H. (2010). Mechanisms underlying inflammation in neurodegeneration. Cell.

[42] Sharma J.N., Al-Omran A., Parvathy S.S. (2007). Role of nitric oxide in inflammatory diseases. Inflammopharmacology.

[43] Wang G., Sun Y., Yang Q., Dai D., Zhang L., Fan H., Zhang W., Dong J., Zhao P. (2023). Liensinine, a alkaloid from lotus plumule, mitigates lipopolysaccharide-induced sepsis-associated encephalopathy through modulation of nuclear factor erythroid 2-related factor-mediated inflammatory biomarkers and mitochondria apoptosis. Food Chem. Toxicol..

[44] Meng X.-L., Zheng L.-C., Liu J., Gao C.-C., Qiu M.-C., Liu Y.-Y., Lu J., Wang D., Chen C.-L. (2017). Inhibitory effects of three bisbenzylisoquinoline alkaloids on lipopolysaccharide-induced microglial activation. RSC Adv..

[45] Zhang L., Gao J., Tang P., Chong L., Liu Y., Liu P., Zhang X., Chen L., Hou C. (2018). Nuciferine inhibits LPS-induced inflammatory response in BV2 cells by activating PPAR-gamma. Int. Immunopharmacol..

[46] Li J., Dong S., Quan S., Ding S., Zhou X., Yu Y., Wu Y., Huang W., Shi Q., Li Q. (2024). Nuciferine reduces inflammation induced by cerebral ischemia-reperfusion injury through the PI3K/Akt/NF-κB pathway. Phytomedicine.

[47] Xie J., Sha T., Tian W., Wu L., Chen J., Huang J., Xia Z., Liu K., Sun P., Fan H. (2023). Anti-inflammatory Activity of Total Alkaloids in Nelumbo nucifera and Simultaneous Determination of Major Bisbenzylisoquinolines. Rev. Bras. De Farmacogn..

[48] Liu T., Zhang L., Joo D., Sun S.-C. (2017). NF-κB signaling in inflammation. Signal Transduct. Target. Ther..

[49] Baeuerle P.A., Baltimore D. (1988). IκB: A specific inhibitor of the NF-κB transcription factor. Science.

[50] Jones S.A., Mills K.H., Harris J. (2013). Autophagy and inflammatory diseases. Immunol. Cell Biol..

[51] Wu M.-Y., Lu J.-H. (2019). Autophagy and macrophage functions: Inflammatory response and phagocytosis. Cells.

[52] Wen X., Wu J., Wang F., Liu B., Huang C., Wei Y. (2013). Deconvoluting the role of reactive oxygen species and autophagy in human diseases. Free. Radic. Biol. Med..

[53] Ye X., Zhu M., Che X., Wang H., Liang X.-J., Wu C., Xue X., Yang J. (2020). Lipopolysaccharide induces neuroinflammation in microglia by activating the MTOR pathway and downregulating Vps34 to inhibit autophagosome formation. J. Neuroinflamm..

[54] Su P., Zhang J., Wang D., Zhao F., Cao Z., Aschner M., Luo W. (2016). The role of autophagy in modulation of neuroinflammation in microglia. Neuroscience.

[55] Deng Z., Zhou X., Lu J.-H., Yue Z. (2021). Autophagy deficiency in neurodevelopmental disorders. Cell Biosci..

[56] Su P., Wu M., Yin X., Li M., Li Y., Bai M., Wang B., Xu E. (2023). Modified Xiaoyao San reverses lipopolysaccharide-induced depression-like behavior through suppressing microglia M1 polarization via enhancing autophagy involved in PI3K/Akt/mTOR pathway in mice. J. Ethnopharmacol..

[57] Petibone D.M., Majeed W., Casciano D.A. (2017). Autophagy function and its relationship to pathology, clinical applications, drug metabolism and toxicity. J. Appl. Toxicol..

[58] Chen S., Guo W., Qi X., Zhou J., Liu Z., Cheng Y. (2019). Natural alkaloids from lotus plumule ameliorate lipopolysaccharide-induced depression-like behavior: Integrating network pharmacology and molecular mechanism evaluation. Food Funct..

[59] Gold P.W., Licinio J., Pavlatou M. (2013). Pathological parainflammation and endoplasmic reticulum stress in depression: Potential translational targets through the CNS insulin, klotho and PPAR-γ systems. Mol. Psychiatry.

[60] Wong V.K., Wu A.G., Wang J.R., Liu L., Law B.Y. (2015). Neferine attenuates the protein level and toxicity of mutant huntingtin in PC-12 cells via induction of autophagy. Molecules.

[61] Wu M.C., Gao Y.H., Zhang C., Ma B.T., Lin H.R., Jiang J.Y., Xue M.F., Li S., Wang H.B. (2023). Liensinine and neferine exert neuroprotective effects via the autophagy pathway in transgenic Caenorhabditis elegans. BMC Complement. Med. Ther..

[62] Yang Z., Goronzy J.J., Weyand C.M. (2015). Autophagy in autoimmune disease. J. Mol. Med..

[63] Jeong J., Choi Y.J., Lee H.K. (2022). The role of autophagy in the function of CD4+ T cells and the development of chronic inflammatory diseases. Front. Pharmacol..

[64] Lapaquette P., Guzzo J., Bretillon L., Bringer M.-A. (2015). Cellular and molecular connections between autophagy and inflammation. Mediat. Inflamm..

[65] Netea-Maier R.T., Plantinga T.S., van de Veerdonk F.L., Smit J.W., Netea M.G. (2016). Modulation of inflammation by autophagy: Consequences for human disease. Autophagy.

[66] Hindle J.V. (2010). Ageing, neurodegeneration and Parkinson’s disease. Age Ageing.

[67] Li Y., Chen Y. (2019). AMPK and autophagy. Autophagy: Biology and Diseases: Basic Science.

[68] Shang L., Wang X. (2011). AMPK and mTOR coordinate the regulation of Ulk1 and mammalian autophagy initiation. Autophagy.

[69] Jeon S.M. (2016). Regulation and function of AMPK in physiology and diseases. Exp. Mol. Med..

[70] Yang P., Zhang H. (2014). You are what you eat: Multifaceted functions of autophagy during C. elegans development. Cell Res..

[71] Jia K., Levine B. (2010). Autophagy and longevity: Lessons from *C. elegans*. Protein Metabolism and Homeostasis in Aging.

[72] Hua F., Li K., Shang S., Wang F., Hu Z. (2019). Immune signaling and autophagy regulation. Autophagy: Biology and Diseases: Basic Science.

[73] Qian M., Fang X., Wang X. (2017). Autophagy and inflammation. Clin. Transl. Med..

[74] Moreira P.I., Smith M.A., Zhu X., Nunomura A., Castellani R.J., Perry G. (2005). Oxidative stress and neurodegeneration. Ann. N. Y. Acad. Sci..

[75] Chatterjee S. (2016). Oxidative stress, inflammation, and disease. Oxidative Stress and Biomaterials.

[76] Ahdab-Barmada M., Moossy J., Nemoto E.M., Lin M.R. (1986). Hyperoxia produces neuronal necrosis in the rat. J. Neuropathol. Exp. Neurol..

[77] Terraneo L., Paroni R., Bianciardi P., Giallongo T., Carelli S., Gorio A., Samaja M. (2017). Brain adaptation to hypoxia and hyperoxia in mice. Redox Biol..

[78] Gandhi S., Abramov A.Y. (2012). Mechanism of oxidative stress in neurodegeneration. Oxidative Med. Cell. Longev..

[79] Rottenberg H., Hoek J.B. (2017). The path from mitochondrial ROS to aging runs through the mitochondrial permeability transition pore. Aging Cell.

[80] Magistretti P.J., Allaman I. (2022). Brain energy and metabolism. Neuroscience in the 21st Century: From Basic to Clinical.

[81] Lin M.T., Beal M.F. (2006). Mitochondrial dysfunction and oxidative stress in neurodegenerative diseases. Nature.

[82] Elfawy H.A., Das B. (2019). Crosstalk between mitochondrial dysfunction, oxidative stress, and age related neurodegenerative disease: Etiologies and therapeutic strategies. Life Sci..

[83] Barber S.C., Mead R.J., Shaw P.J. (2006). Oxidative stress in ALS: A mechanism of neurodegeneration and a therapeutic target. Biochim. Et Biophys. Acta (BBA)-Mol. Basis Dis..

[84] Lenaz G., Bovina C., D’aurelio M., Fato R., Formiggini G., Genova M.L., Giuliano G., Pich M.M., Paolucci U., Castelli G.P. (2002). Role of mitochondria in oxidative stress and aging. Ann. N. Y. Acad. Sci..

[85] Beckman J.S., Koppenol W.H. (1996). Nitric oxide, superoxide, and peroxynitrite: The good, the bad, and ugly. Am. J. Physiol.-Cell Physiol..

[86] Bayazeid O., Nemutlu E., Eylem C.C., Yalcin F.N. (2020). Neuroactivity of naturally occurring proaporphine alkaloid, pronuciferine. J. Biochem. Mol. Toxicol..

[87] Limón-Pacheco J., Gonsebatt M.E. (2009). The role of antioxidants and antioxidant-related enzymes in protective responses to environmentally induced oxidative stress. Mutat. Res./Genet. Toxicol. Environ. Mutagen..

[88] Goc Z., Szaroma W., Kapusta E., Dziubek K. (2017). Protective effects of melatonin on the activity of SOD, CAT, GSH-Px and GSH content in organs of mice after administration of SNP. Chin. J. Physiol..

[89] Zhu X., Hao R., Lv X., Su J., Li D., Zhang C. (2024). Neuroprotective effects of nuciferine on high-fat diet-induced cognitive dysfunction in obese mice: Role of insulin resistance, neuroinflammation, and oxidative stress. Food Front..

[90] Yuan Y., Cruzat V.F., Newsholme P., Cheng J., Chen Y., Lu Y. (2016). Regulation of SIRT1 in aging: Roles in mitochondrial function and biogenesis. Mech. Ageing Dev..

[91] Kadenbach B. (2012). Introduction to mitochondrial oxidative phosphorylation. Mitochondrial Oxidative Phosphorylation: Nuclear-Encoded Genes, Enzyme Regulation, and Pathophysiology.

[92] Tauffenberger A., Magistretti P.J. (2021). Reactive oxygen species: Beyond their reactive behavior. Neurochem. Res..

[93] Kausar S., Wang F., Cui H. (2018). The role of mitochondria in reactive oxygen species generation and its implications for neurodegenerative diseases. Cells.

[94] Stefanatos R., Sanz A. (2018). The role of mitochondrial ROS in the aging brain. FEBS Lett..

[95] Kim G.J., Chandrasekaran K., Morgan W.F. (2006). Mitochondrial dysfunction, persistently elevated levels of reactive oxygen species and radiation-induced genomic instability: A review. Mutagenesis.

[96] Bhat A.H., Dar K.B., Anees S., Zargar M.A., Masood A., Sofi M.A., Ganie S.A. (2015). Oxidative stress, mitochondrial dysfunction and neurodegenerative diseases; a mechanistic insight. Biomed. Pharmacother..

[97] Alqahtani T., Deore S.L., Kide A.A., Shende B.A., Sharma R., Chakole R.D., Nemade L.S., Kale N.K., Borah S., Deokar S.S. (2023). Mitochondrial dysfunction and oxidative stress in Alzheimer’s disease, and Parkinson’s disease, Huntington’s disease and amyotrophic lateral sclerosis-an updated review. Mitochondrion.

[98] Nasr P., Gursahani H.I., Pang Z., Bondada V., Lee J., Hadley R.W., Geddes J.W. (2003). Influence of cytosolic and mitochondrial Ca^2+^, ATP, mitochondrial membrane potential, and calpain activity on the mechanism of neuron death induced by 3-nitropropionic acid. Neurochem. Int..

[99] Schon E.A., Manfredi G. (2003). Neuronal degeneration and mitochondrial dysfunction. J. Clin. Investig..

[100] Wu C., Chen J., Yang R., Duan F., Li S., Chen X. (2019). Mitochondrial protective effect of neferine through the modulation of nuclear factor erythroid 2-related factor 2 signalling in ischaemic stroke. Br. J. Pharmacol..

[101] Qian S., Wei Z., Yang W., Huang J., Yang Y., Wang J. (2022). The role of BCL-2 family proteins in regulating apoptosis and cancer therapy. Front. Oncol..

[102] Hashem K.S., Elkelawy A.M.M.H., Abd-Allah S., Helmy N.A. (2020). Involvement of Mfn2, Bcl2/Bax signaling and mitochondrial viability in the potential protective effect of Royal jelly against mitochondria-mediated ovarian apoptosis by cisplatin in rats. Iran. J. Basic Med. Sci..

[103] Zhang Q., Liu J., Duan H., Li R., Peng W., Wu C. (2021). Activation of Nrf2/HO-1 signaling: An important molecular mechanism of herbal medicine in the treatment of atherosclerosis via the protection of vascular endothelial cells from oxidative stress. J. Adv. Res..

[104] Zorov D.B., Juhaszova M., Sollott S.J. (2014). Mitochondrial reactive oxygen species (ROS) and ROS-induced ROS release. Physiol. Rev..

[105] Orfali R., Alwatban A.Z., Orfali R.S., Lau L., Chea N., Alotaibi A.M., Nam Y.-W., Zhang M. (2024). Oxidative stress and ion channels in neurodegenerative diseases. Front. Physiol..

[106] Cordaro M., Cuzzocrea S., Di Paola R. (2022). Ion channels and neurodegenerative disease aging related. Ion Transporters-From Basic Properties to Medical Treatment.

[107] Olsen M.L., Khakh B.S., Skatchkov S.N., Zhou M., Lee C.J., Rouach N. (2015). New insights on astrocyte ion channels: Critical for homeostasis and neuron-glia signaling. J. Neurosci..

[108] Arundine M., Tymianski M. (2003). Molecular mechanisms of calcium-dependent neurodegeneration in excitotoxicity. Cell Calcium.

[109] Cojocaru A., Burada E., Bălșeanu A.-T., Deftu A.-F., Cătălin B., Popa-Wagner A., Osiac E. (2021). Roles of microglial ion channel in neurodegenerative diseases. J. Clin. Med..

[110] Urrutia J., Arrizabalaga-Iriondo A., Sanchez-del-Rey A., Martinez-Ibargüen A., Gallego M., Casis O., Revuelta M. (2024). Therapeutic role of voltage-gated potassium channels in age-related neurodegenerative diseases. Front. Cell. Neurosci..

[111] Zündorf G., Reiser G. (2011). Calcium dysregulation and homeostasis of neural calcium in the molecular mechanisms of neurodegenerative diseases provide multiple targets for neuroprotection. Antioxid. Redox Signal..

[112] Verma M., Wills Z., Chu C.T. (2018). Excitatory dendritic mitochondrial calcium toxicity: Implications for Parkinson’s and other neurodegenerative diseases. Front. Neurosci..

[113] Mattson M.P. (2002). Oxidative stress, perturbed calcium homeostasis, and immune dysfunction in Alzheimer’s disease. J. Neurovirology.

[114] Simunkova M., Alwasel S.H., Alhazza I.M., Jomova K., Kollar V., Rusko M., Valko M. (2019). Management of oxidative stress and other pathologies in Alzheimer’s disease. Arch. Toxicol..

[115] Meng X.L., Liu S.Y., Xue J.S., Gou J.M., Wang D., Liu H.S., Chen C.L., Xu C.B. (2022). Protective effects of Liensinine, Isoliensinine, and Neferine on PC12 cells injured by amyloid-beta. J. Food Biochem..

[116] Kurt M., Davies D., Kidd M., Duff K., Howlett D. (2003). Hyperphosphorylated tau and paired helical filament-like structures in the brains of mice carrying mutant amyloid precursor protein and mutant presenilin-1 transgenes. Neurobiol. Dis..

[117] Kong L., Yang J., Yang H., Xu B., Yang T., Liu W. (2024). Research advances on CaMKs-mediated neurodevelopmental injury. Arch. Toxicol..

[118] Sinsky J., Pichlerova K., Hanes J. (2021). Tau Protein Interaction Partners and Their Roles in Alzheimer’s Disease and Other Tauopathies. Int. J. Mol. Sci..

[119] Sengking J., Oka C., Yawoot N., Tocharus J., Chaichompoo W., Suksamrarn A., Tocharus C. (2022). Protective effect of Neferine in permanent cerebral ischemic rats via anti-oxidative and anti-apoptotic mechanisms. Neurotox. Res..

[120] Kuznetsova L., Basova N., Shpakov A. (2023). Neuronal NO Synthase in the Pathogenesis of Metabolic Syndrome. Cell Tissue Biol..

[121] Tellios V. (2021). The Role of Neuronal Nitric Oxide Synthase in Regulating Cerebellar Network Formation Across Murine Development. Ph.D. Thesis.

[122] Wen J., Li H., Zhang Y., Li X., Liu F. (2015). Modification of HSP proteins and Ca^2+^ are responsible for the NO-derived peroxynitrite mediated neurological damage in PC12 cell. Int. J. Clin. Exp. Pathol..

[123] Albakova Z., Mangasarova Y., Albakov A., Gorenkova L. (2022). HSP70 and HSP90 in cancer: Cytosolic, endoplasmic reticulum and mitochondrial chaperones of tumorigenesis. Front. Oncol..

[124] Wang K.K. (2000). Calpain and caspase: Can you tell the difference?. Trends Neurosci..

[125] Sayre L.M., Zelasko D.A., Harris P.L., Perry G., Salomon R.G., Smith M.A. (1997). 4-Hydroxynonenal-derived advanced lipid peroxidation end products are increased in Alzheimer’s disease. J. Neurochem..

[126] Culig L., Chu X., Bohr V.A. (2022). Neurogenesis in aging and age-related neurodegenerative diseases. Ageing Res. Rev..

[127] Polis B., Samson A.O. (2021). Neurogenesis versus neurodegeneration: The broken balance in Alzheimer’s disease. Neural Regen. Res..

[128] Abdipranoto A., Wu S., Stayte S., Vissel B. (2008). The role of neurogenesis in neurodegenerative diseases and its implications for therapeutic development. CNS Neurol. Disord.-Drug Targets (Former. Curr. Drug Targets—CNS Neurol. Disord.).

[129] Horgusluoglu E., Nudelman K., Nho K., Saykin A.J. (2017). Adult neurogenesis and neurodegenerative diseases: A systems biology perspective. Am. J. Med. Genet. Part B Neuropsychiatr. Genet..

[130] Davinelli S., Medoro A., Ali S., Passarella D., Intrieri M., Scapagnini G. (2023). Dietary flavonoids and adult neurogenesis: Potential implications for brain aging. Curr. Neuropharmacol..

[131] Johnson G., Jope R. (1992). The role of microtubule-associated protein 2 (MAP-2) in neuronal growth, plasticity, and degeneration. J. Neurosci. Res..

[132] Matus A. (1991). Microtubule-associated proteins and neuronal morphogenesis. J. Cell Sci..

[133] Aleksandr S., Alexey G. (2022). Role of the MBP protein in myelin formation and degradation in the brain. Biol. Commun..

[134] Kister A., Kister I. (2023). Overview of myelin, major myelin lipids, and myelin-associated proteins. Front. Chem..

[135] Zhu J.J., Yu B.Y., Huang X.K., He M.Z., Chen B.W., Chen T.T., Fang H.Y., Chen S.Q., Fu X.Q., Li P.J. (2021). Neferine Protects against Hypoxic-Ischemic Brain Damage in Neonatal Rats by Suppressing NLRP3-Mediated Inflammasome Activation. Oxidative Med. Cell. Longev..

[136] Lee Y.J., Lin C.M., Chang Y.C., Yang M.Y., Wang C.J., Hsu L.S. (2024). Nelumbo nucifera leaves extract ameliorated scopolamine-induced cognition impairment via enhanced adult hippocampus neurogenesis. Environ. Toxicol..

[137] Bayazeid O., Nemutlu E., Eylem C.C., Ilhan M., Kupeli-Akkol E., Karahan H., Kelicen-Ugur P., Ersoz T., Yalcin F.N. (2021). Neuroactivity of the naturally occurring aporphine alkaloid, roemerine. Nat. Prod. Res..

[138] Yang T., Nie Z., Shu H., Kuang Y., Chen X., Cheng J., Yu S., Liu H. (2020). The role of BDNF on neural plasticity in depression. Front. Cell. Neurosci..

[139] Popova N.K., Ilchibaeva T.V., Naumenko V.S. (2017). Neurotrophic Factors (BDNF and GDNF) and the Serotonergic System of the Brain. Biochemistry.

[140] Azman K.F., Zakaria R. (2022). Recent advances on the role of brain-derived neurotrophic factor (BDNF) in neurodegenerative diseases. Int. J. Mol. Sci..

[141] Colucci-D’Amato L., Speranza L., Volpicelli F. (2020). Neurotrophic factor BDNF, physiological functions and therapeutic potential in depression, neurodegeneration and brain cancer. Int. J. Mol. Sci..

[142] Dworkin S., Mantamadiotis T. (2010). Targeting CREB signalling in neurogenesis. Expert Opin. Ther. Targets.

[143] Ortega-Martínez S. (2015). A new perspective on the role of the CREB family of transcription factors in memory consolidation via adult hippocampal neurogenesis. Front. Mol. Neurosci..

[144] Baldessarini R.J., Tarazi F.I. (1996). Brain dopamine receptors: A primer on their current status, basic and clinical. Harv. Rev. Psychiatry.

[145] Kawahata I., Finkelstein D.I., Fukunaga K. (2024). Dopamine D1–D5 receptors in brain nuclei: Implications for Health and Disease. Receptors.

[146] Ramesh S., Arachchige A.S.P.M. (2023). Depletion of dopamine in Parkinson’s disease and relevant therapeutic options: A review of the literature. AIMS Neurosci..

[147] Hisahara S., Shimohama S. (2011). Dopamine Receptors and Parkinson′ s Disease. Int. J. Med. Chem..

[148] Anzalone A., Lizardi-Ortiz J.E., Ramos M., De Mei C., Hopf F.W., Iaccarino C., Halbout B., Jacobsen J., Kinoshita C., Welter M. (2012). Dual control of dopamine synthesis and release by presynaptic and postsynaptic dopamine D2 receptors. J. Neurosci..

[149] Isaacson S.H., Hauser R.A., Pahwa R., Gray D., Duvvuri S. (2023). Dopamine agonists in Parkinson’s disease: Impact of D1-like or D2-like dopamine receptor subtype selectivity and avenues for future treatment. Clin. Park. Relat. Disord..

[150] Jing S., Wang Z., Zhang J., Li X., Huang R. (2021). Neuroprotective effect of neferine, an alkaloid against the 1-methyl-4-phenyl-1, 2, 3, 6-tetrahydropyridine induced Parkinson’s disease mouse model. Pharmacogn. Mag..

[151] Hardy J., Selkoe D.J. (2002). The amyloid hypothesis of Alzheimer’s disease: Progress and problems on the road to therapeutics. Science.

[152] Yang Z., Song Z., Xue W., Sheng J., Shu Z., Shi Y., Liang J., Yao X. (2014). Synthesis and structure–activity relationship of nuciferine derivatives as potential acetylcholinesterase inhibitors. Med. Chem. Res..

[153] Jung H.A., Karki S., Kim J.H., Choi J.S. (2015). BACE1 and cholinesterase inhibitory activities of Nelumbo nucifera embryos. Arch. Pharmacal Res..

[154] Alharbi B., Al-Kuraishy H.M., Al-Gareeb A.I., Elekhnawy E., Alharbi H., Alexiou A., Papadakis M., Batiha G.E.-S. (2024). Role of GABA pathway in motor and non-motor symptoms in Parkinson’s disease: A bidirectional circuit. Eur. J. Med. Res..

[155] Bi D., Wen L., Wu Z., Shen Y. (2020). GABAergic dysfunction in excitatory and inhibitory (E/I) imbalance drives the pathogenesis of Alzheimer’s disease. Alzheimer’s Dement..

[156] Menéndez-Perdomo I.M., Facchini P.J. (2018). Benzylisoquinoline Alkaloids Biosynthesis in Sacred Lotus. Molecules.

[157] Bennett M.R., Thompson M.L., Shepherd S.A., Dunstan M.S., Herbert A.J., Smith D.R.M., Cronin V.A., Menon B.R.K., Levy C., Micklefield J. (2018). Structure and Biocatalytic Scope of Coclaurine *N*-Methyltransferase. Angew. Chem. Int. Ed..

[158] Weng T., Shen C., Chiu Y., Lin Y., Huang Y. (2012). Effects of armepavine against hepatic fibrosis induced by thioacetamide in rats. Phytother. Res..

[159] Wei X., Zhang M., Yang M., Ogutu C., Li J., Deng X. (2024). Lotus (*Nelumbo nucifera*) benzylisoquinoline alkaloids: Advances in chemical profiling, extraction methods, pharmacological activities, and biosynthetic elucidation. Veg. Res..

[160] Yang M., Zhu L., Li L., Li J., Xu L., Feng J., Liu Y. (2017). Digital gene expression analysis provides insight into the transcript profile of the genes involved in aporphine alkaloid biosynthesis in lotus (*Nelumbo nucifera*). Front. Plant Sci..

[161] Kempster P., Ma A. (2022). Parkinson’s disease, dopaminergic drugs and the plant world. Front. Pharmacol..

[162] Ye L.-H., He X.-X., You C., Tao X., Wang L.-S., Zhang M.-D., Zhou Y.-F., Chang Q. (2018). Pharmacokinetics of nuciferine and N-nornuciferine, two major alkaloids from *Nelumbo nucifera* leaves, in rat plasma and the brain. Front. Pharmacol..

[163] Gilgun-Sherki Y., Melamed E., Offen D. (2001). Oxidative stress induced-neurodegenerative diseases: The need for antioxidants that penetrate the blood brain barrier. Neuropharmacology.

[164] Bhambhani S., Kondhare K.R., Giri A.P. (2021). Diversity in chemical structures and biological properties of plant alkaloids. Molecules.

[165] Cheng Y., Li H.-L., Zhou Z.-W., Long H.-Z., Luo H.-Y., Wen D.-D., Cheng L., Gao L.-C. (2021). Isoliensinine: A Natural Compound with “Drug-Like” Potential. Front. Pharmacol..

[166] Poornima P., Weng C.F., Padma V.V. (2014). Neferine, an alkaloid from lotus seed embryo, inhibits human lung cancer cell growth by MAPK activation and cell cycle arrest. Biofactors.

[167] Cabedo N., Berenguer I., Figadere B., Cortes D. (2009). An overview on benzylisoquinoline derivatives with dopaminergic and serotonergic activities. Curr. Med. Chem..

[168] Plazas E., Muñoz D.R. (2022). Natural isoquinoline alkaloids: Pharmacological features and multi-target potential for complex diseases. Pharmacol. Res..

[169] Portoghese P.S. (1970). Relationships between stereostructure and pharmacological activities. Annu. Rev. Pharmacol..

[170] Yang G.-M., Sun J., Pan Y., Zhang J.-L., Xiao M., Zhu M.-S. (2018). Isolation and identification of a tribenzylisoquinoline alkaloid from *Nelumbo nucifera* Gaertn, a novel potential smooth muscle relaxant. Fitoterapia.

[171] Jung H.A., Jin S.E., Choi R.J., Kim D.H., Kim Y.S., Ryu J.H., Son Y.K., Park J.J., Choi J.S. (2010). Anti-amnesic activity of neferine with antioxidant and anti-inflammatory capacities, as well as inhibition of ChEs and BACE1. Life Sci..

[172] Yu Y., Sun S., Wang S., Zhang Q., Li M., Lan F., Li S., Liu C. (2016). Liensinine- and Neferine-Induced Cardiotoxicity in Primary Neonatal Rat Cardiomyocytes and Human-Induced Pluripotent Stem Cell-Derived Cardiomyocytes. Int. J. Mol. Sci..

[173] Deng W., Li H., Zhang Y., Lin Y., Chen C., Chen J., Huang Y., Zhou Y., Tang Y., Ding J. (2023). Isoliensinine suppresses bone loss by targeted inhibition of RANKL-RANK binding. Biochem. Pharmacol..

[174] Dabrell S.N., Li Y.-C., Yamaguchi H., Chen H.-F., Hung M.-C. (2023). Herbal Compounds Dauricine and Isoliensinine Impede SARS-CoV-2 Viral Entry. Biomedicines.

[175] Ma C., Wang J., Chu H., Zhang X., Wang Z., Wang H., Li G. (2014). Purification and characterization of aporphine alkaloids from leaves of *Nelumbo nucifera* Gaertn and their effects on glucose consumption in 3T3-L1 adipocytes. Int. J. Mol. Sci..

[176] Kim S.-M., Park E.-J., Lee H.-J. (2022). Nuciferine attenuates lipopolysaccharide-stimulated inflammatory responses by inhibiting p38 MAPK/ATF2 signaling pathways. Inflammopharmacology.

