# Supplementary material for: Neuroprotective Potential of Major Alkaloids from Nelumbo nucifera (Lotus): Mechanisms and Therapeutic Implications
Source: Int J Mol Sci. 2025 Aug 26;26(17):8280. doi: 10.3390/ijms26178280 (PMC12428744; doi:10.3390/ijms26178280)
Supplement: Supplementary file 1 [file ijms-26-08280-s001.zip › ijms-3795611-supplementary.pdf]

International Journal of *Molecular Sciences*

MDPI

Supplementary materials

Neuroprotective Potential of Major Alkaloids from *Nelumbo nucifera* (Lotus): Mechanisms and Therapeutic Implications

Douyang Zhao [1], Linlin Ma [1,2], Jeremy Brownlie [2], Kathryn Tonissen [1,2], Yang Pan [3] and Yunjiang Feng [1,2,*]

[1] Institute for Biomedicine and Glycomics, Griffith University, Brisbane 4111 Australia; douyang.zhao@griffithuni.edu.au (D.Z.); linlin.ma@griffith.edu.au (L.M.); k.tonissen@griffith.edu.au (K.F.T.)
[2] School of Environment and Science, Griffith University, Brisbane 4111, Australia; j.brownlie@griffith.edu.au
[3] School of Pharmacy, Nanjing University of Chinese Medicine, Nanjing 21023, China; ypan@njucm.edu.cn
* Correspondence: y.feng@griffith.edu.au

1 1-Benzylisoquinoline alkaloids

1-Benzylisoquinoline alkaloids (**1–15**) (Figure S1) are present in trace amounts in *N. nucifera*. Several, including coclaurine and armepavine, are also found in other medicinal plants [1-3]. Common substituents include hydroxyl or methoxy groups located at C-6 and C-7 of the isoquinoline ring, and an additional substitution R_3 on the 4-hydroxyphenyl ring. An exception to this structure is norjuziphine (**14**), where the R_1 and R_2 substituents are located at C-7 and C-8, instead of the typical C-6 and C-7 positions.

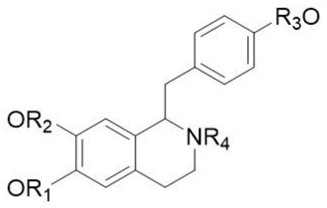

1-11 1-benzylisoquinoline

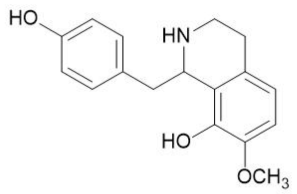

12 Norjuziphine

		R_1	R_2	R_3	R_4
**1**	Norcoclaurine	H	H	H	H
**2**	Coclaurine	CH_3	H	H	H
**3**	*N*-Methylcoclaurine	CH_3	H	H	CH_3
**4**	Norarmepavine	CH_3	CH_3	H	H
**5**	*N*-Methylisococlaurine	H	CH_3	H	CH_3
**6**	6-demethyl-4’-O-methylN-methylcoclaurine	H	H	CH_3	CH_3
**7**	Armepavine	CH_3	CH_3	H	CH_3
**8**	4′-O-Methyl-N-methylcoclaurine	CH_3	H	H	CH_3
**9**	4′-O-Methylarmepavine	CH_3	CH_3	CH_3	CH_3
**10**	Lotusine	H	CH_3	H	$N^+(CH_3)_2$
**11**	Bromo, methyl armepavine	CH_3	CH_3	Br	CH_3
**12**	Methoxy, methyl isoquinolinol	CH_3	CH_3	H	CH_3
**13**	Higenamine-glucoside	H	H	Glu	H

Figure S1. 1-Benzylisoquinoline alkaloids identified in lotus.

2. Aporphine alkaloids

Aporphine alkaloids (16–36) (Figure S2) were mainly present in the leaves, with nuciferine (21) being the most abundant [4, 5]. These aporphine alkaloids can be categorized into four structural variations based on their structural modifications. Compounds 16–24 represent the basic aporphines, possessing a classical aporphine scaffold with variable substituents (e.g., hydroxyl, methoxy, and nitrogen) at the R1, R2, and R3 positions. Compounds 25–30 are classified as dehydroaporphines with dehydrogenation or carbonyl substitution on the A and B rings. Compounds 31–32 belong to the oxoaporphine with a carbonyl group integrated into the aporphine ring system, leading to a more oxidized structure. Compounds 33–35 represent N-oxide aporphines, featuring *N*-oxidation at the nitrogen atom, which contributes to altered electronic properties and potential shifts in bioactivity. Pronuciferine (36) is a highly modified aporphine featuring two methoxy groups, *N*-methylation, and a ketone moiety, indicating significant structural divergence.

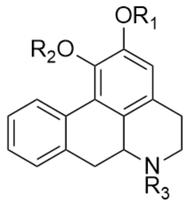

16-24 aporphine

		R_1	R_2	R_3
**16**	Caaverine	CH_3	H	H
**17**	Asimilobine	H	CH_3	H
**18**	Lirinidine	CH_3	H	CH_3
**19**	*O*-Nornuciferine	H	CH_3	CH_3
**20**	*N*-Nornuciferine	CH_3	CH_3	H
**21**	Nuciferine	CH_3	CH_3	CH_3
**22**	Anonaine	-CH_2-		H
**23**	Roemerine	-CH_2-		CH_3
**24**	*N*-methylasimilobine	H	CH_3	CH_3

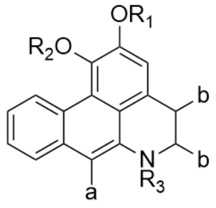

25-30 aporphine

		R_1	R_2	R_3	a	b
**25**	Dehydroanonaine	-CH_2-	H			
**26**	Dehydroroemerine	-CH_2-		CH_3		
**27**	7-Hydroxydehydronuciferine	CH_3	CH_3	CH_3	OH	
**28**	Dehydronuciferine	CH_3	CH_3	CH_3		
**29**	Dehydroaporphine	H	CH_3	H		
**30**	Cepharadione	CH_3	CH_3	CH3		=O

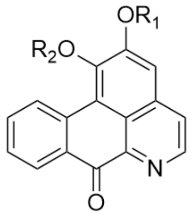

31-32 aporphine

		R_1	R_2
**31**	Lysicamine	CH_3	CH_3
**32**	Liriodenine	-CH_2-	

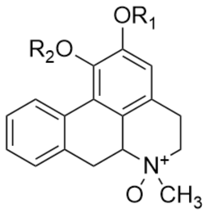

33-35 aporphine

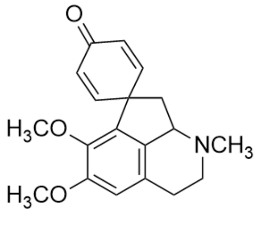

36 Pronuciferine

		R_1	R_2
**33**	*N*-methyl asimilobine-N-oxide	H	CH_3
**34**	Roemerine-N-oxide	-CH_2-	
**35**	Nuciferine-N-oxide	CH_3	CH_3

Figure S2. Aporphine alkaloids identified in lotus.

Notably, many aporphine alkaloids possess dopamine-like structures, allowing them to cross the blood-brain barrier and interact with dopaminergic signalling pathways [6, 7]. For instance, N-nornuciferine (20) has been shown to exhibit favorable pharmacokinetics and rapid brain penetration following systemic administration [8]. Given the restrictive nature of the blood-brain barrier, endogenous antioxidant defenses within neurons and glial cells are critical for neuroprotection, and alkaloids with antioxidant potential may offer significant therapeutic value [9].

3. Bisbenzylisoquinoline alkaloids

Bisbenzylisoquinoline alkaloids (37–50) (Figure S3) mainly accumulate in seed embryos and are the most abundant alkaloids [4, 10]. Among the alkaloids, liensinine (38), isoliensinine (39), and neferine (40) are the most abundant bisbenzylisoquinoline alkaloids, with yields of 2.57%, 4.38%, and 9.25% of the total alkaloid content, respectively [11, 12]. Bisbenzylisoquinoline alkaloids are characterized by a dimeric structure comprising two 1-benzylisoquinoline units linked by ether linkages. Their large, hydrophobic, and flexible frameworks enable them to interact with diverse biological targets, such as calcium channels, serotonin receptors, and mitochondria, contributing to their multifunctional biological roles [13, 14].

The primary substituents on the bisbenzylisoquinoline framework typically include hydroxyl or methoxy groups positioned at the R1, R2, R3, and R4 sites, which correspond to various locations on the isoquinoline rings and their benzyl counterparts. These substituents modulate the polarity, conformational dynamics, and biological activity of the compound. Methyl neferine (43) and negferine (44) were highly methylated, while compounds 45–50 exhibited isomeric relationships. These stereochemical features can significantly influence receptor-binding affinity, membrane interactions, and overall pharmacological profiles [15].

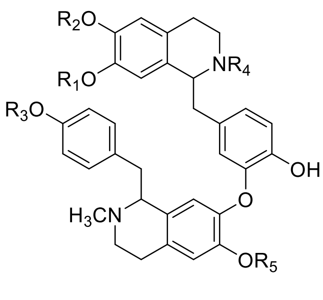

37-44 bisbenzylisoquinioline

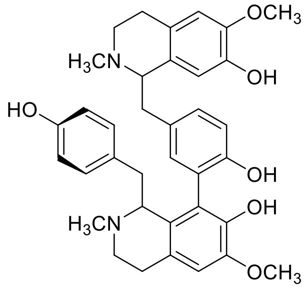

45 Nelumborine B

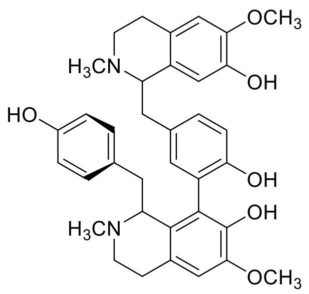

46 Nelumborine A

		R$_1$	R$_2$	R$_3$	R$_4$	R$_5$
**37**	Nelumboferine	H	CH_3	H	CH_3	H
**38**	Liensinine	H	CH_3	CH_3	CH_3	H
**39**	Isoliensinine	CH_3	H	CH_3	CH_3	H
**40**	Neferine	CH_3	CH_3	CH_3	CH_3	H
**41**	6-Hydroxynorisoliensinine	H	CH_3	H	CH_3	H
**42**	N-Norisoliensinine	H	CH_3	CH_3	H	H
**43**	Methyl neferine	CH_3	CH_3	CH_3	CH_3	CH_3
**44**	Negferine	CH_3	CH_3	H	CH_3	CH_3

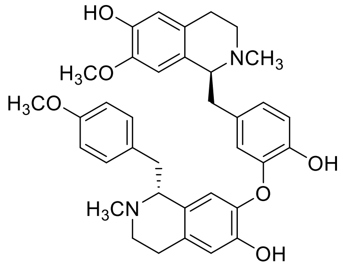

47 1'-epi-isoliensinine

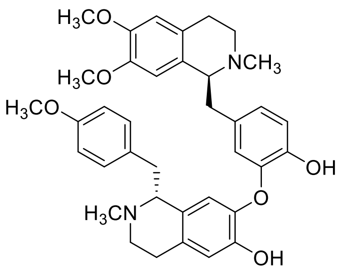

48 1'-epi-neferine

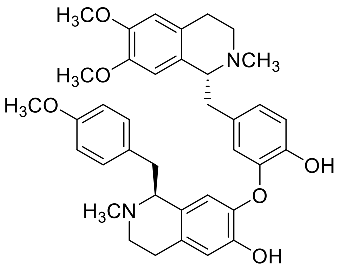

49 1-epi-neferine

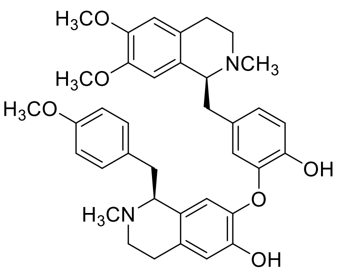

50 ent-neferine

Figure S3. Bisbenzylisoquinoline alkaloids identified in lotus.

4. Tribenzylisoquinoline alkaloids

Neoliensinine (51) (Figure S4) was the only tribenzylisoquinoline alkaloid isolated from N. nucifera. This compound features three 1-benzylisoquinoline units connected via two ether linkages, resulting in a rigid, symmetrical macrocyclic structure. Recent studies have demonstrated its activity as a muscle relaxant, suggesting possible applications in cardiovascular therapy [16].

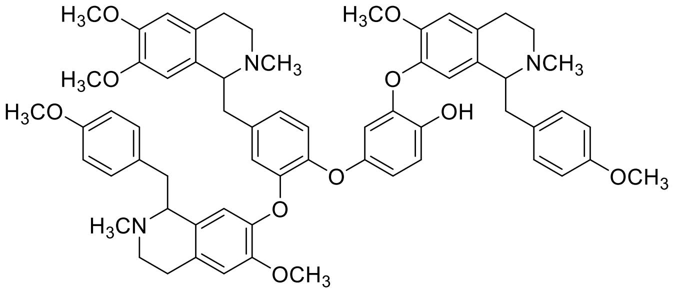

51 Neoliensinine

Figure S4. Tribenzylisoquinoline alkaloid neoliensinine identified in lotus.

Table S1. Cytotoxicity of lotus alkaloids.

**Alkaloid**	**Cell Type**	**Non-cytotoxic Range**	**Notes**	**Reference**
Neferine	RAW 264.7 mouse macrophages	≤ 30 µM	Marginal cytotoxicity observed at higher doses	[17]
	BEAS-2B normal human lung cells	≤ 20 µM	No significant suppression of cell growth	[12]
	Neonatal rat cardiomyocytes	0.12 – 10 µM	No cytotoxicity	[18]
Liensinine	Neonatal rat cardiomyocytes	0.12 – 10 µM	No cytotoxicity	[18]
Isoliensinine	Bone marrow macrophages	< 7.5 µM	No cytotoxicity or proliferation effects	[19]
	HEK-293T human embryonic kidney cells	≤ 10 µM	No cytotoxicity	[20]
Pronuciferine	SH-SY5Y human neuroblastoma cells	≤ 100 µM	No toxicity	[21]
	3T3-L1 adipocytes	≤ 5 µg/mL	No cytotoxicity	[22]
Nuciferine	RAW 264.7 mouse macrophages	≤ 50 µM	No cytotoxicity	[23]
	3T3-L1 adipocytes	≤ 2 µg/mL	No obvious cytotoxicity	[22]
Roemerine	3T3-L1 adipocytes	≤ 2 µg/mL	No obvious cytotoxicity	[22]

Reference

1. Menéndez-Perdomo, I.M.; Facchini, P.J. Benzylisoquinoline Alkaloids Biosynthesis in Sacred Lotus. *Molecules* **2018**, *23*, 2899.
2. Bennett, M.R.; Thompson, M.L.; Shepherd, S.A.; Dunstan, M.S.; Herbert, A.J.; Smith, D.R.M.; Cronin, V.A.; Menon, B.R.K.; Levy, C.; Micklefield, J. Structure and Biocatalytic Scope of Coclaurine *N*-Methyltransferase. *Angew. Chem. Int. Ed.* **2018**, *57*, 10600–10604.
3. Weng, T.; Shen, C.; Chiu, Y.; Lin, Y.; Huang, Y. Effects of armepavine against hepatic fibrosis induced by thioacetamide in rats. *Phytother. Res.* **2012**, *26*, 344–353.
4. Wei, X.; Zhang, M.; Yang, M.; Ogutu, C.; Li, J.; Deng, X. Lotus (*Nelumbo nucifera*) benzylisoquinoline alkaloids: Advances in chemical profiling, extraction methods, pharmacological activities, and biosynthetic elucidation. *Veg. Res.* **2024**, *4*, e005.
5. Yang, M.; Zhu, L.; Li, L.; Li, J.; Xu, L.; Feng, J.; Liu, Y. Digital gene expression analysis provides insight into the transcript profile of the genes involved in aporphine alkaloid biosynthesis in lotus (*Nelumbo nucifera*). *Front. Plant Sci.* **2017**, *8*, 80.

6. Zhou, H.; Hou, T.; Gao, Z.; Guo, X.; Wang, C.; Wang, J.; Liu, Y.; Liang, X. Discovery of eight alkaloids with D1 and D2 antagonist activity in leaves of Nelumbo nucifera Gaertn. Using FLIPR assays. *J. Ethnopharmacol.* **2021**, *278*, 114335.
7. Kempster, P.; Ma, A. Parkinson's disease, dopaminergic drugs and the plant world. *Front. Pharmacol.* **2022**, *13*, 970714.
8. Ye, L.-H.; He, X.-X.; You, C.; Tao, X.; Wang, L.-S.; Zhang, M.-D.; Zhou, Y.-F.; Chang, Q. Pharmacokinetics of nuciferine and N-nornuciferine, two major alkaloids from *Nelumbo nucifera* leaves, in rat plasma and the brain. *Front. Pharmacol.* **2018**, *9*, 902.
9. Gilgun-Sherki, Y.; Melamed, E.; Offen, D. Oxidative stress induced-neurodegenerative diseases: the need for antioxidants that penetrate the blood brain barrier. *Neuropharmacology* **2001**, *40*, 959–975.
10. Bhambhani, S.; Kondhare, K.R.; Giri, A.P. Diversity in chemical structures and biological properties of plant alkaloids. *Molecules* **2021**, *26*, 3374.
11. Cheng, Y.; Li, H.-L.; Zhou, Z.-W.; Long, H.-Z.; Luo, H.-Y.; Wen, D.-D.; Cheng, L.; Gao, L.-C. Isoliensinine: A Natural Compound with "Drug-Like" Potential. *Front. Pharmacol.* **2021**, *12*, 630385.
12. Poornima, P.; Weng, C.F.; Padma, V.V. Neferine, an alkaloid from lotus seed embryo, inhibits human lung cancer cell growth by MAPK activation and cell cycle arrest. *Biofactors* **2014**, *40*, 121–131.
13. Cabedo, N.; Berenguer, I.; Figadere, B.; Cortes, D. An overview on benzylisoquinoline derivatives with dopaminergic and serotonergic activities. *Curr. Med. Chem.* **2009**, *16*, 2441–2467.
14. Plazas, E.; Muñoz, D.R. Natural isoquinoline alkaloids: Pharmacological features and multi-target potential for complex diseases. *Pharmacol. Res.* **2022**, *177*, 106126.
15. Portoghese, P.S. Relationships between stereostructure and pharmacological activities. *Annu. Rev. Pharmacol.* **1970**, *10*, 51–76.
16. Yang, G.-M.; Sun, J.; Pan, Y.; Zhang, J.-L.; Xiao, M.; Zhu, M.-S. Isolation and identification of a tribenzylisoquinoline alkaloid from *Nelumbo nucifera* Gaertn, a novel potential smooth muscle relaxant. *Fitoterapia* **2018**, *124*, 58–65.
17. Jung, H.A.; Jin, S.E.; Choi, R.J.; Kim, D.H.; Kim, Y.S.; Ryu, J.H.; Son, Y.K.; Park, J.J.; Choi, J.S. Anti-amnesic activity of neferine with antioxidant and anti-inflammatory capacities, as well as inhibition of ChEs and BACE1. *Life Sci.* **2010**, *87*, 420–430.
18. Yu, Y.; Sun, S.; Wang, S.; Zhang, Q.; Li, M.; Lan, F.; Li, S.; Liu, C. Liensinine- and Neferine-Induced Cardiotoxicity in Primary Neonatal Rat Cardiomyocytes and Human-Induced Pluripotent Stem Cell-Derived Cardiomyocytes. *Int. J. Mol. Sci.* **2016**, *17*, 186.
19. Deng, W.; Li, H.; Zhang, Y.; Lin, Y.; Chen, C.; Chen, J.; Huang, Y.; Zhou, Y.; Tang, Y.; Ding, J.; et al. Isoliensinine suppresses bone loss by targeted inhibition of RANKL-RANK binding. *Biochem. Pharmacol.* **2023**, *210*, 115463.
20. Dabrell, S.N.; Li, Y.-C.; Yamaguchi, H.; Chen, H.-F.; Hung, M.-C. Herbal Compounds Dauricine and Isoliensinine Impede SARS-CoV-2 Viral Entry. *Biomedicines* **2023**, *11*, 2914.
21. Goc, Z.; Szaroma, W.; Kapusta, E.; Dziubek, K. Protective effects of melatonin on the activity of SOD, CAT, GSH-Px and GSH content in organs of mice after administration of SNP. Chin. J. Physiol. **2017**, *60*, 1–10.
22. Ma, C.; Wang, J.; Chu, H.; Zhang, X.; Wang, Z.; Wang, H.; Li, G. Purification and characterization of aporphine alkaloids from leaves of *Nelumbo nucifera* Gaertn and their effects on glucose consumption in 3T3-L1 adipocytes. *Int. J. Mol. Sci.* **2014**, *15*, 3481–3494.
23. Kim, S.-M.; Park, E.-J.; Lee, H.-J. Nuciferine attenuates lipopolysaccharide-stimulated inflammatory responses by inhibiting p38 MAPK/ATF2 signaling pathways. *Inflammopharmacology* **2022**, *30*, 2373–2383.
